# Genomic evidence for recurrent genetic admixture during the domestication of Mediterranean olive trees (*Olea europaea* L.)

**DOI:** 10.1186/s12915-020-00881-6

**Published:** 2020-10-26

**Authors:** Irene Julca, Marina Marcet-Houben, Fernando Cruz, Jèssica Gómez-Garrido, Brandon S. Gaut, Concepción M. Díez, Ivo G. Gut, Tyler S. Alioto, Pablo Vargas, Toni Gabaldón

**Affiliations:** 1 Centre for Genomic Regulation (CRG), The Barcelona Institute of Science and Technology, Dr. Aiguader 88, 08003 Barcelona, Spain; 2 Department of Experimental and Health Sciences, Universitat Pompeu Fabra (UPF), 08003 Barcelona, Spain; 3 Universitat Autònoma de Barcelona (UAB), 08193 Barcelona, Spain; 4 Present address: Barcelona Supercomputing Centre (BSC-CNS), and Institute for Research in Biomedicine (IRB), Barcelona, Spain; 5 CNAG-CRG, Centre for Genomic Regulation (CRG), Barcelona Institute of Science and Technology (BIST), Baldiri i Reixac 4, 08028 Barcelona, Spain; 6 Department Ecology and Evolutionary Biology, University of California Irvine, Irvine, CA 92697 USA; 7 Agronomy Department, University of Cordoba, 14071 Cordoba, Spain; 8 Royal Botanical Garden of Madrid. Consejo Superior de Investigaciones Científicas (CSIC), 28014 Madrid, Spain; 9 Institució Catalana de Recerca i Estudis Avançats (ICREA), Pg. Lluís Companys 23, 08010 Barcelona, Spain

**Keywords:** Admixture, Domestication, Genome, Introgression, Olive

## Abstract

**Background:**

Olive tree (*Olea europaea L. subsp*. *europaea*, Oleaceae) has been the most emblematic perennial crop for Mediterranean countries since its domestication around 6000 years ago in the Levant. Two taxonomic varieties are currently recognized: cultivated (var. *europaea*) and wild (var. *sylvestris*) trees. However, it remains unclear whether olive cultivars derive from a single initial domestication event followed by secondary diversification, or whether cultivated lineages are the result of more than a single, independent primary domestication event. To shed light into the recent evolution and domestication of the olive tree, here we analyze a group of newly sequenced and available genomes using a phylogenomics and population genomics framework.

**Results:**

We improved the assembly and annotation of the reference genome, newly sequenced the genomes of twelve individuals: ten var. *europaea*, one var. *sylvestris*, and one outgroup taxon (subsp. *cuspidata*)—and assembled a dataset comprising whole genome data from 46 var. *europaea* and 10 var. *sylvestris*. Phylogenomic and population structure analyses support a continuous process of olive tree domestication, involving a major domestication event, followed by recurrent independent genetic admixture events with wild populations across the Mediterranean Basin. Cultivated olives exhibit only slightly lower levels of genetic diversity than wild forms, which can be partially explained by the occurrence of a mild population bottleneck 3000–14,000 years ago during the primary domestication period, followed by recurrent introgression from wild populations. Genes associated with stress response and developmental processes were positively selected in cultivars, but we did not find evidence that genes involved in fruit size or oil content were under positive selection. This suggests that complex selective processes other than directional selection of a few genes are in place.

**Conclusions:**

Altogether, our results suggest that a primary domestication area in the eastern Mediterranean basin was followed by numerous secondary events across most countries of southern Europe and northern Africa, often involving genetic admixture with genetically rich wild populations, particularly from the western Mediterranean Basin.

## Background

The Mediterranean olive tree (*Olea europaea L. subsp*. *europaea*, Oleaceae) is one of the earliest cultivated fruit trees of the Mediterranean Basin (MB). Current classifications recognize two taxonomic varieties of *O. europaea*: var. *sylvestris* (hereafter *sylvestris*, also named oleaster) for wild populations and var. *europaea* (hereafter *europaea*) for cultivated forms [1, 2]. Both varieties are predominantly out-crossing and have long lifespans, including a long juvenile phase that can last up to 15 years in natural conditions. The natural distribution of the Mediterranean olive encompasses all countries of the MB, although a few wild populations have also been found in northern areas with low occurrence of frost [3]. Cultivars have historically been planted nearby wild populations since ancient times, where they exchange pollen that have resulted in effective crop production and historical hybridization [4].

There is a large body of evidence pointing to the eastern MB as the cradle of the first domestication event of olives. According to archeological, palaeobotanical, and genetic studies, the crop was domesticated from eastern wild progenitors around 6000 years ago [5–8]. Once superior individuals were selected, clonal propagation made their multiplication and the spread of valuable agronomic traits possible. Clonal propagation also facilitated the spread of cultivars from the eastern to the western MB via classical civilizations such as Phoenicians, Greeks, or Romans. After six millennia, olive domestication has resulted in a vast number of cultivars of uncertain pedigree that are often geographically restricted [9].

It remains unclear whether olive cultivars derived from a single initial domestication event in the Levant, followed by secondary diversification [8, 10, 11], or whether cultivated lineages are the result of more than a single, independent primary domestication event [7, 12–16]. Previous studies based on plastid and nuclear markers have suggested controversial but not necessarily incompatible domestication scenarios. The reconstruction of plastid lineages has yielded unresolved phylogenies due to low plastid diversity [8, 17, 18]. For instance, 90% of olive cultivars across the MB share the same “eastern like” plastid haplotype [18]. This general result is congruent with archeological data [5], which suggests a major domestication event in the Levant, possibly followed by recurrent admixture events with local wild olives that would have contributed to the crop diversity. However, nuclear markers showed a more complex pattern. Olive cultivars clustered together into three different gene pools, with a rough geographical correspondence to the eastern, central, and western MB [12, 19–22]. The relationship between these groups also showed two interesting features [12]. First, the western group (southern Spain and Portugal) retained the fingerprint of a genetic bottleneck and, surprisingly, was closely related to cultivated accessions from the Levant [12]. Second, the central MB group, which also included the cultivars from eastern Spain (Catalonia, Valencia, and Balearic Islands), showed signals of recent and extensive admixture with local wild populations and relatively high plastid diversity compared to the other groups of cultivars. These differential patterns, along with the fact that many cultivars from the central MB retain wild-like phenotypic characteristics, opened the controversial question of a possible minor domestication center for olives in the central MB [10, 14]. Recently, the analysis of the genome of a set of traditional olive cultivars and carbonized pit remains from Spanish Roman archeological sites and has favored this latter hypothesis. However, the phylogenomic and population genomic analyses applied ignored plastid lineages and some other important features of domestication [16].

Approximate Bayesian computing (ABC) models have been applied to infer the demographic history of olives and were consistent with a primary domestication event in the East [8, 12]. These models also highlighted the paramount role of admixture to account for the diversity of the crop. This feature was particularly predominant in cultivars from the central MB, where ~ 20% of the genetic diversity of olives may have been acquired via introgression with local wild populations [12]. So far, genetic and archeological sources of evidence have agreed with the existence of a major center of domestication for olives in the Levant, but questions remain about the extent of admixture and the potential for secondary centers of domestication elsewhere in the MB. To gain novel insights into this open question and into the most recent evolution of the cultivated olive tree, we sequenced twelve accessions, including ten representative cultivars, one wild individual of var. *sylvestris*, and one individual from *O. europaea* subsp. *cuspidata* to be used as a distant outgroup in our analysis (Table 1). The ten cultivars were carefully selected to capture the signal of the divergent genetic clusters reported by previous studies among the cultivated olive germplasm [8, 12]. Together, they represent the following: (i) the three genetic pools identified in the MB (West: “Picual” and “Lechin de Granada;” Central: “Arbequina,” “Frantoio,” “Koroneiki,” and “Megaritiki;” East: “Beladi” and “Sorani”); (ii) the main plastid lineages found in the cultivated olive (“Lechin de Sevilla” and “Megaritiki”—E2.3 and E2.2, respectively; “Chemlal de Kabilye”—E3.2; the rest of cultivars—E1.1); and (iii) the main cultivar diversity from the most important areas of Mediterranean olive cultivation. For instance, “Arbequina” is the most international cultivar due to its adaptation to high-density planting designs, “Picual” covers more than 1.5 million of hectares in southern Spain, “Frantoio” and “Koroneiki” are the primary cultivars in Italy and Greece, respectively, and “Beladi” and “Sorani” are widely used in the Levant. Of note, our selection includes only authenticated cultivars preserved in the germplasm collection of Cordoba University—i.e., those that matched by DNA and morphological markers with authentic control samples from its natural area of origin and/or cultivation [9]. For *sylvestris*, previous phylogenetic results and field experience led us to choose a wild individual from an isolated area in order to avoid potential feral or highly introgressed trees. Hence, we sampled a tree from a location by the coast near the Cantabrian mountains (northern Spain) where the olive tree has not been historically cultivated and that is 200 km distant from current plantations. These populations had previously been screened using fingerprinting techniques [3] and Sanger sequencing [18].

**Table 1:** *O. europaea* genomes used in the analysis. The columns show the sample origin, analysis in which it was used, plastid group [17], nuclear group [12], ploidy level, and the source of the data. *Cultivars that are duplicated and are excluded from the main analysis (see “Methods”)

*O. europaea* subsp*.*	Origin	Analysis	Plastid group	Nuclear group	Ploidy level	Source
*europaea* var. *europaea* cv. “Farga”	Spain (Boadilla/La Senia)	Nuclear, plastid, mitochondrial	E3.1	Central MB (Q2)	2x	ENA (PRJEB4992)
*europaea* var. *europaea* cv. “Arbequina”	Spain (BGMO_UCO)	Nuclear, plastid, mitochondrial	E1.1	Central MB (Q2)	2x	This study
*europaea* var. *europaea* cv. “Picual”	Spain (BGMO_UCO)	Nuclear, plastid, mitochondrial	E1.1	Western MB (Q1)	2x	This study
*europaea* var. *europaea* cv. “Beladi”	Lebanon (BGMO_UCO)	Nuclear, plastid, mitochondrial	E1.1	Eastern MB (Q3)	2x	This study
*europaea* var. *europaea* cv. “Sorani”	Syria (BGMO_UCO)	Nuclear, plastid, mitochondrial	E1.1	Eastern MB (Q3)	2x	This study
*europaea* var. *europaea* cv. “Manzanilla”	Spain	Plastid	E1.1	–	2x	NCBI (FN996972)
*europaea* var*. europaea* cv. “Bianchera”	Italy	Plastid	E1.1	–	2x	NCBI (NC_013707)
*europaea* var. *europaea* cv. “Chemlal de Kabilye”	Algeria (BGMO_UCO)	Nuclear, plastid, mitochondrial	E3.2	Mosaic (Q2 + Q1)	2x	This study
*europaea* var. *europaea* cv. “Megaritiki”	Greece (BGMO_UCO)	Nuclear, plastid, mitochondrial	E2.2	Central (Q2)	2x	This study
*europaea* var. *europaea* cv. “Lechin de Sevilla”	Spain (BGMO_UCO)	Nuclear, plastid, mitochondrial	E2.3	Mosaic (Q1 + Q2)	2x	This study
*europaea* var. *europaea* cv. “Lechin de Granada”	Spain (BGMO_UCO)	Nuclear, plastid, mitochondrial	E1.1	Western MB (Q1)	2x	This study
*europaea* var. *europaea* cv. “Frantoio”	Italy (BGMO_UCO)	Nuclear, plastid, mitochondrial	E1.1	Central (Q2)	2x	This study
*europaea* var. *europaea* cv. “Koroneiki”	Greece (BGMO_UCO)	Nuclear, plastid, mitochondrial	E1.1	Central (Q2)	2x	This study
*europaea* var. *sylvestris “sylvestris*-T*”*	Turkey	Nuclear, plastid, mitochondrial	E1.1	–	2x	NCBI (PRJNA417827)
*europaea* var. *sylvestris “sylvestris*-S*”*	Spain (Pechón)	Nuclear, plastid, mitochondrial	E3	–	2x	This study
*europaea* var. *sylvestris* “W7R224”	Morocco	Nuclear, plastid, mitochondrial	E3	–	2x	SRR9860491
*europaea* var. *sylvestris* “W11R37”	Spain	Nuclear, plastid, mitochondrial	E3	–	2x	SRR9860496
*europaea* var. *sylvestris* “W9R302”	Albania	Nuclear, plastid, mitochondrial	E2	–	2x	SRR9860497
*europaea* var. *sylvestris* “W8R225”	Morocco	Nuclear, plastid, mitochondrial	E1	–	2x	SRR9860498
*europaea* var. *sylvestris* “W4R183”	Spain	Nuclear, plastid, mitochondrial	E1	–	2x	SRR9860503
*europaea* var. *sylvestris* “W3R78”	Spain (Menorca island)	Nuclear, plastid, mitochondrial	E2	–	2x	SRR9860505
*europaea* var. *sylvestris* “W2R74”	Spain	Nuclear, plastid, mitochondrial	E3	–	2x	SRR9860532
*europaea* var. *sylvestris* “W1R198”	Croatia	Nuclear, plastid, mitochondrial	E2	–	2x	SRR9860533
*europaea* var. *europaea* cv. “Myrtolia”	Greece	Nuclear, plastid, mitochondrial	E1	–	2x	SRR9860492
*europaea* var. *europaea* cv. “Klon-14-1812”	Albania	Nuclear, plastid, mitochondrial	E1	–	2x	SRR9860493
*europaea* var. *europaea* cv. “Barri”	Syria	Nuclear, plastid, mitochondrial	E1	–	2x	SRR9860494
*europaea* var. *europaea* cv. “Grappolo”	Italy	Nuclear, plastid, mitochondrial	E1	–	2x	SRR9860495
*europaea* var. *europaea* cv. “Mari”	Iran	Nuclear, plastid, mitochondrial	E1	–	2x	SRR9860499
*europaea* var. *europaea* cv. “Lianolia Kerkyras”	Greece	Nuclear, plastid, mitochondrial	E2	–	2x	SRR9860500
*europaea* var. *europaea* cv. “Piñonera”	Spain	Nuclear, plastid, mitochondrial	E1	–	2x	SRR9860501
*europaea* var. *europaea* cv. “Mavreya”	Greece	Nuclear, plastid, mitochondrial	E1	–	2x	SRR9860502
*europaea* var. *europaea* cv. “Royal”	Spain	Nuclear, plastid, mitochondrial	E1	–	2x	SRR9860504
*europaea* var. *europaea* cv. “Llumeta”	Spain	Nuclear, plastid, mitochondrial	E3	–	2x	SRR9860506
*europaea* var. *europaea* cv. “Barnea”	Israel	Nuclear, plastid, mitochondrial	E1	–	2x	SRR9860507
*europaea* var. *europaea* cv. “Majhol-1013”	Syria	Nuclear, plastid, mitochondrial	E1	–	2x	SRR9860508
*europaea* var. *europaea* cv. “Mastoidis”	Greece	Nuclear, plastid, mitochondrial	E1	–	2x	SRR9860509
*europaea* var. *europaea* cv. “Abbadi Abou Gabra-842”	Syria	Nuclear, plastid, mitochondrial	E1	–	2x	SRR9860510
*europaea* var. *europaea* cv. “Fishomi”	Iran	Nuclear, plastid, mitochondrial	E1	–	2x	SRR9860511
*europaea* var. *europaea* cv. “Abou Satl Mohazam”	Syria	Nuclear, plastid, mitochondrial	E1	–	2x	SRR9860512
*europaea* var. *europaea* cv. “Forastera de Tortosa”	Spain	Nuclear, plastid, mitochondrial	E3	–	2x	SRR9860514
*europaea* var. europaea cv. “Dokkar”	Tunisia	Nuclear, plastid, mitochondrial	E3	–	2x	SRR9860516
*europaea* var. europaea cv. “Jabali”	Syria	Nuclear, plastid, mitochondrial	E1	–	2x	SRR9860517
*europaea* var. *europaea* cv. “Majhol-152”	Syria	Nuclear, plastid, mitochondrial	E1	–	2x	SRR9860518
*europaea* var. *europaea* cv. “Picudo”	Spain	Nuclear, plastid, mitochondrial	E1	–	2x	SRR9860519
*europaea* var. *europaea* cv. “Maarri”	Syria	Nuclear, plastid, mitochondrial	E1	–	2x	SRR9860520
*europaea* var. *europaea* cv. “Temprano”	Spain	Nuclear, plastid, mitochondrial	E2	–	2x	SRR9860521
*europaea* var. *europaea* cv. “Menya”	Spain	Nuclear, plastid, mitochondrial	E2	–	2x	SRR9860522
*europaea* var. *europaea* cv. “Koroneiki”*	Greece	Nuclear, plastid, mitochondrial	E1.1	–	2x	SRR9860523
*europaea* var. *europaea* cv. “Manzanilla de Sevilla”	Spain	Nuclear, plastid, mitochondrial	E1	–	2x	SRR9860524
*europaea* var. *europaea* cv. “Uslu”	Turkey	Nuclear, plastid, mitochondrial	E1	–	2x	SRR9860525
*europaea* var. *europaea* cv. “Lechin de Sevilla”*	Spain	Nuclear, plastid, mitochondrial	E2.3	–	2x	SRR9860526
*europaea* var. *europaea* cv. “Ocal”	Spain	Nuclear, plastid, mitochondrial	E1	–	2x	SRR9860527
*europaea* var. *europaea* cv. “Abou Kanani”	Syria	Nuclear, plastid, mitochondrial	E1	–	2x	SRR9860528
*europaea* var. *europaea* cv. “Verdial de Velez-Malaga-1”	Spain	Nuclear, plastid, mitochondrial	E1	–	2x	SRR9860529
*europaea* var. *europaea* cv. “Arbequina”*	Spain	Nuclear, plastid, mitochondrial	E1.1	–	2x	SRR9860530
*europaea* var. *europaea* cv. “Chemlal de Kabylie”*	Algeria	Nuclear, plastid, mitochondrial	E3.2	–	2x	SRR9860531
*europaea* var. *europaea* cv. “Leccino”	Italy	Nuclear, plastid, mitochondrial	E1	–	2x	SRR9860534
*europaea* var. *europaea* cv. “Manzanillera de Huercal Overa”	Spain	Nuclear, plastid, mitochondrial	E2	–	2x	SRR9860535
*europaea* var. *europaea* cv. “Hojiblanca”	Spain	Nuclear, plastid, mitochondrial	E1	–	2x	SRR9860536
*europaea* var. *europaea* cv. “Kalamon”	Greece	Nuclear, plastid, mitochondrial	E1	–	2x	SRR9860537
*europaea* var. *europaea* cv. “Picual”*	Spain	Nuclear, plastid, mitochondrial	E1.1	–	2x	SRR9860538
*europaea* var. *europaea* cv. “Zarza”	Spain	Nuclear, plastid, mitochondrial	E2	–	2x	SRR9860539
*europaea* var. *europaea* cv. “Morrut”	Spain	Nuclear, plastid, mitochondrial	E1	–	2x	SRR9860540
*europaea* var. *europaea* cv. “Frantoio”*	Italy	Nuclear, plastid, mitochondrial	E1.1	–	2x	SRR9860541
*europaea* var. *sylvestris* “Stavrovouni 11”	Cyprus	Plastid	E1.4	–	2x	NCBI (HF558645)
*europaea var. sylvestris “Haut Atlas 1”*	Morocco (High Atlas)	Plastid	E2	–	2x	NCBI (NC_015401)
*europaea* var. *sylvestris* “Gue de Constantine 20”	Algeria: Gue de Constantine, Algiers	Plastid	E3	–	2x	NCBI (FN997651)
*europaea* var*. sylvestris* “Oeiras 1”	Portugal	Plastid	E3.1	–	2x	NCBI (MG255763)
*europaea* var. *sylvestris* “Vallee du Fango 5”	France	Plastid	E2.1	–	2x	NCBI (MG255762)
*maroccana* “Immouzzer S1”	Morocco (High Atlas)	Plastid	M	–	6x	NCBI (NC_015623)
*guanchica* “La Gomera 10”	Spain	Plastid	M-g1	–	2x	NCBI (MG255764)
*laperrinei* “Adjelella 10”	Algeria	Plastid	E1-l1	–	2x	NCBI (MG255765)
*cuspidata* - R	Reunion island	Nuclear, plastid, mitochondrial	A	–	2x	This study
*cuspidata* “Almihwit 5.1”	Yemen	Plastid	C2	–	2x	NCBI (FN996943)
*cuspidata* “Guanghzou 1”	China	Plastid	C1	–	2x	NCBI (FN996944)
*cuspidata* “Maui 1”	USA (Hawaii-Maui)	Plastid	A	–	2x	NCBI (NC_015604)
*cuspidata* “Menagesha Forest 14”	Ethiopia	Plastid	C2	–	2x	NCBI (MG255760)

These twelve newly obtained sequences plus 43 additional sequences published in a recent study [16] complement the three available genomes for the species: the cv. Farga from eastern Spain, for which we provide here an improved assembly by anchoring it to a genetic map; the cv. Picual; and an oleaster (var. *sylvestris*) from Turkey [16, 23, 24]. These datasets combined constitute the largest and most comprehensive ensemble of olive whole genomic information, which we analyzed here under a phylogenomics and population genomics framework to shed light on the recent evolution and domestication of the olive tree. In particular, we addressed the question whether genome-wide variation data can disentangle scenarios of one versus multiple centers of domestication. Additionally, we were interested in finding out whether genetic introgression between wild and cultivated trees have historically played a role in the domestication process, to assess the presence of potential domestication bottlenecks and to identify genes and genomic regions under selection. Finally, sequencing of nuclear genomes allowed testing earlier suggestions of close relationship between cultivars of distant locations such as southern Spain and the Levant [12].

## Results

### New assembly version of the reference olive (cultivar Farga) genome

We improved the available *O. europaea* var. *europaea* (cv. Farga) genome assembly (Oe6 version) [23] by anchoring it to chromosomes using a publicly available genetic map [25] and removing 201 scaffolds which likely represent contaminating sequences (see “Methods”). In the final assembly (Oe9), 520.5 Mbp (39.5%) of Oe6 sequence was anchored to 23 linkage groups, of which 288 Mbp (21.8%) were oriented. This anchored assembly (Oe9) has a higher N50 (734,380) than the recently published assembly for cv. Picual (410,451) [16], but lower than the assembly for *O. europaea* var. *sylvestris* (12,567,911) [24]. Nevertheless, Oe9 is much less fragmented than the *sylvestris* genome, displaying a ~ 5-fold reduction in the number of scaffolds (9751 vs 41,261 scaffolds), and has larger scaffolds than the cv. Picual genome (Additional file 1: Fig. S1 and Additional file 2: Table S1). The comparison between Oe9 and *sylvestris* assemblies (Additional file 1: Fig. S1b) shows a high level of conserved synteny and many regions appeared duplicated between the two genomes. These duplicated syntenic regions showed different levels of divergence, with Ks values peaking around median values of 0.32 and 59.19. This supports the occurrence of more than one ancient polyploidization event in *O. europaea*, as proposed earlier [26].

Additionally, we improved the genome annotation in Oe9 genome, by extended automated functional annotation and by manual curation of some of the genes. Conserved gene completeness results for both Oe6 and Oe9 support that the decontamination step (see “Methods”) did not result in the loss of sequences coding for important plant genes. Moreover, the Oe9 genome assembly has clearly higher gene completeness than the *sylvestris* reference (93% vs 87%). The cvs. Picual and Farga (Oe9) assemblies have very similar gene completeness values, although the percentage of duplicated sequence is twice in cv. Picual (Additional file 2: Table S1). To better characterize duplicated sequence differences between the cvs. Farga (Oe9) and Picual references, two RNAseq libraries (SRR6003535, ERR1406351) were aligned to the two genome assemblies. Results confirmed that both assemblies are very similar in terms of completeness but show a high percentage of multi-mappings in the cv. Picual reference (Additional file 2: Table S2), which suggests the presence of some artefactual duplications in the Picual reference.

The Oe9 assembly has 4911 more annotated genes than the var. *sylvestris* genome. A comparison of gene sets using BLASTN [27] with identity > 80% and *e*-value < 1e−5 cutoffs shows that 5245 Oe9 annotated genes do not have a match in *sylvestris*, and conversely, 2620 genes of *sylvestris* do not have a match in *europaea* (Oe9). Distinct genome annotation methods could partly explain these differences [23, 24]. To have an annotation-independent measure of the differences between both assemblies, we mapped raw reads of both genome projects to the alternative assembly and assessed coverage of the putative unique genes (see “Methods”). We first filtered out the genes that have at least 50% of their length with a read coverage higher than 20, which resulted in 2115 and 280 unique genes for *europaea* and *sylvestris*, respectively. Even when lowering the coverage threshold to 5, *europaea* still had more unique genes (1756) than *sylvestris* (102). Of these, we discarded 131 Oe9-specific genes as possible contaminations as their first BLAST hit fell outside plants. Thus, some of the genes uniquely found in the Oe9 assembly may represent true differences in terms of gene content. We assessed the presence of these genes in all the other samples and found that roughly 50% of those genes were not found in any of them (1092). We removed 104 Oe9 unique genes that appeared clustered into contigs with no other genes and had blast hits in other non-plant genomes, potentially indicating remaining contamination. The remaining Oe9 unique genes are often found in clusters within larger contigs, indicating they may have been lost together. Among the 1023 genes that are absent from the *sylvestris* genome and are found in Oe9 and at least one of the other cultivars, there are functions associated with stress response, such as HIPPs (heavy metal-associated isoprenylated plant proteins) [28], LEA (late embryogenesis abundant) [29], and salicylic acid-binding [30]. Other genes are associated with growth and development. This is the case of RALF (rapid alkalinization factor), which has been shown to arrest root growth and development in tomato and *Arabidopsis* [31], and caffeoyl shikimate esterase (CSE), which is an enzyme central to the lignin biosynthetic pathway [32]. It is well known that lignin biosynthesis contributes to plant growth, tissue and organ development, and response to a variety of biotic and abiotic stresses [33]. Some other Oe9 unique genes were associated with seed dormancy and sugar signaling, DOG1 [34], and positive regulation of germination, PELPK1 [35] (see Additional file 2: Table S3). Only two *sylvestris* unique genes had annotated functions (Additional file 2: Table S3). One corresponds to GSH-induced LITAF, which negatively regulates hypersensitive cell death in *Arabidopsis* [36]. The other one corresponds to FAR1 (far-red-impaired response)-related sequence, with roles in diverse developmental and physiological processes [37, 38].

In addition, we assembled the plastid and mitochondrial genomes of the cv. Farga, which were not provided as separate assemblies in the previous release [23] (see “Methods”). The final assembly of the plastid genome comprised 155,658 base pairs (bp) (Additional file 2: Table S4), in agreement with previously reported olive plastid sequences, which range from 155,531 to 155,896 bp [17, 39, 40]. We annotated 130 genes out of the 130–133 genes reported for other olive plastid genomes (see Additional file 1: Fig. S2, Additional file 2: Table S4) [17, 39], of which 85 are protein coding genes, 37 are transfer RNAs, and eight are ribosomal RNAs. The final assembly of the mitochondrial genome has a size of 755,572 bp (Additional file 2: Table S4), which is similar to that of previously sequenced wild olive mitochondrial genomes (710,737–769,995 bp) [40]. The coding regions in the olive mitochondrion comprise 46 protein-coding genes, 3 ribosomal RNA genes, and 26 transfer RNA genes (Additional file 1: Fig. S3, Additional file 2: Table S4).

### Contrasting genetic diversity patterns in organellar and nuclear genomes

**Fig. 1:**
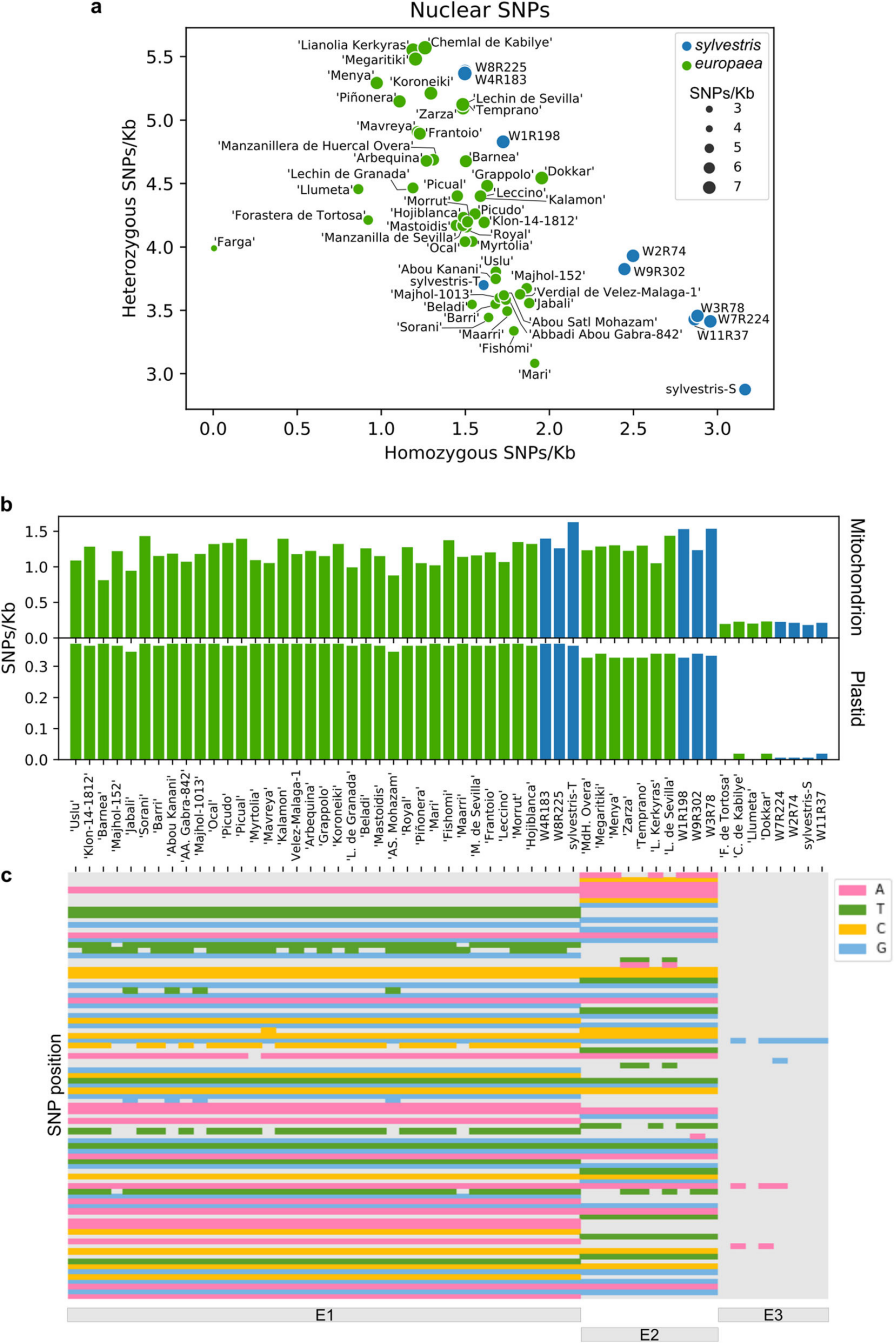
SNP density (SNPs/kb) in sequenced individuals. **a** Homozygous versus heterozygous SNPs for each accession, relative to the cv. Farga reference. Dot size correlates with the total amount of SNPs. All the cultivars are marked in green and var. *sylvestris* in blue. **b** SNP densities for the plastid and mitochondrial genomes. **c** Plot showing the relative position and identity of plastid SNPs compared to the cv. Farga (reference genome). Bars on the bottom show the main plastid haplotypes of the individuals as described by Besnard et al. [17, 44]

We used this improved reference genome assembly (Oe9) to call SNPs at the nuclear, plastid, and mitochondrial genomes for all subsp. *europaea* individuals for which whole genome sequence information is available. For this, we compiled data comprising a total of 56 unique subsp. *europaea* individuals, combining samples sequenced in this project and samples from recent publications [16, 23, 24] (see “Methods”, Table 1). This new dataset included 46 different cultivars and 10 individuals described as var. *sylvestris*. Altogether, for wild and cultivated olives (subsp. *europaea*), we obtained a total of 24,724,756 polymorphic positions uniformly distributed along the nuclear genome (Additional file 1: Fig. S4 and S5), 85 in the plastid genome, and 3979 in the mitochondrial genome (see Additional file 1: Fig. S2, S3). In the plastid genome, a large region (~ 25 kb) was found to be fully conserved and devoid of SNPs in all analyzed individuals (Additional file 1: Fig. S2). This region includes the largest plastid gene, ycf2, which has also been found to exhibit low rates of nucleotide substitution in other plants [41]. This gene is essential for plant survival; however, its exact function is unknown [42, 43]. This conserved region also comprises other genes, including ycf15, rps7, rpl2, ndhB, rRNA, and tRNA. All individuals presented similar amounts of nuclear polymorphisms relative to the Oe9 reference (Fig. 1a). Interestingly, the *sylvestris* from northern Spain (*sylvestris*-S) has a higher number of homozygous SNPs and a lower number of heterozygous SNPs (Fig. 1a). The other *sylvestris* have different patterns, five of them (W2R74, W9R302, W3R78, W7R224, W11R37) have a high number of homozygous SNPs, but similar number of heterozygous SNPs as some cultivars, while the other four *sylvestris* (W8R225, W4R183, W1R198, *sylvestris*-T) have a number of homozygous and heterozygous SNPs in the range of variation found in cultivars (Fig. 1a).

Strikingly, the relative number of organellar polymorphisms for the different individuals did not follow the patterns observed in the nuclear genome (Fig. 1b). The plastid and mitochondrial genomes of four *sylvestris* (W7R224, W2R74, W11R37, and *sylvestris*-S) and four cvs. (Chemlal de Kabilye, Forastera de Tortosa, Llumeta, and Dokkar) show a notably lower number of SNPs relative to the cv. Farga reference genome (Fig. 1b). Sequence variation in the plastid was remarkably low, in agreement with previous studies [17]. Plastid genomes can be arranged into three groups based on nucleotide polymorphisms, which are congruent with the three main plastid lineages (E1, E2, and E3) already described (Fig. 1c) [17, 44]. More interestingly, eight individuals, four cultivars and four oleasters, share the same haplotype as cv. Farga (E3), with 3 or less SNPs of difference (Fig. 1c). Hence, the nuclear and organellar genomes presumably reflect different evolutionary histories. Notably, the organellar genomes of the cvs. Farga, Chemlal de Kabilye, Forastera de Tortosa, Llumeta, and Dokkar, and the wild individuals W7R224, W2R74, W11R37, and *sylvestris*-S, all from the western MB, show a very close genetic relationship (Fig. 1b, c). As organelles are maternally inherited in olive [45], our results suggest that these cultivars and wild individuals share a recent common maternal ancestor. Similarly, “Megaritiki” (from Greece), “Lianolia Kerkyras” (Greece), “Temprano” (Spain), “Menya” (Spain), Manzanillera de Huercal Overa (Spain), “Zarza” (Spain), and “Lechin de Sevilla” (Spain) share the same plastid haplotype (E2) as the *sylvestris* W1R198 (from Croatia), W9R302 (Albania), W3R78 (Spain), which can also be found in wild populations but exclusively from central and western Mediterranean areas [18]. The other cultivars and the three oleasters (W4R183, W8R225, *sylvestri*s-T) share the E1 haplotype.

Furthermore, we found a very close relationship at the organellar and nuclear levels between the eastern cultivars from Syria, Iran, Lebanon, and Turkey (e.g., “Sorani,” “Jabali,” “Beladi,” “Fishomi,” “Mari,” and “Uslu”) and cultivars from southern Spain (e.g., “Picual,” “Lechín de Granada,” “Manzanilla de Sevilla,” “Hojiblanca,” and “Picudo”). This finding provides support for a previously proposed hypothesis of a recent bottleneck affecting a subset of western cultivars [12]. This “local” bottleneck affected only western olive cultivars (mostly from southern Spain and Portugal), and it was probably related to the introduction of olive germplasm into southern Spain during the Muslim period. This period began c. 700 AD, lasted eight centuries, and possibly reshaped the cultivated olive germplasm of the Iberian Peninsula due to purported migration of cultivars from the Levant and the possible substitution of the previously cultivated local germplasm [16].

### Population structure and phylogenetic relationships of the olive tree

**Fig. 2:**
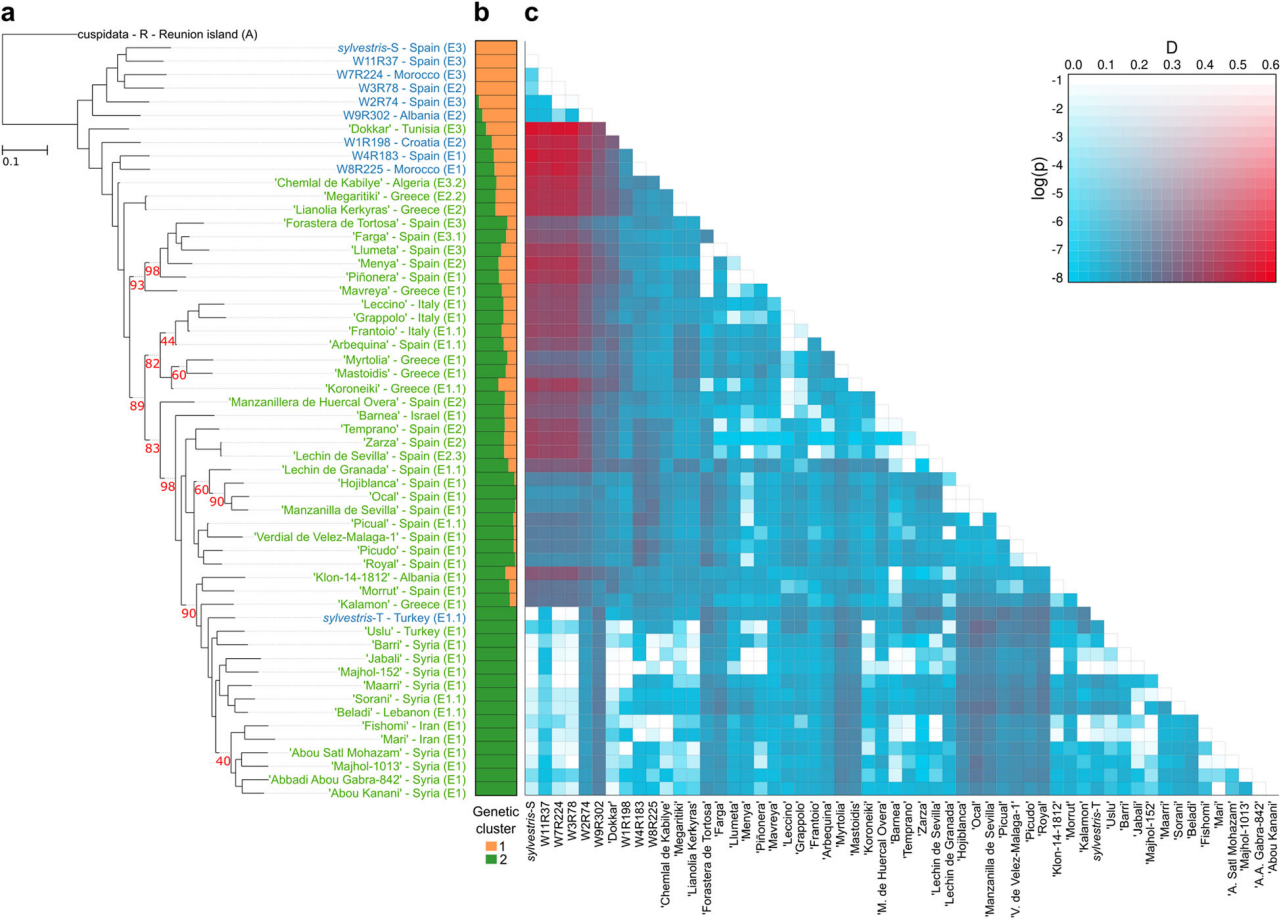
Maximum likelihood species tree derived from the nuclear SNPs data. **a** Nuclear phylogeny. Cultivated olives are shown in green and wild olives in blue. The geographical location of the accession and the plastid haplotype are indicated. Only bootstrap values below 100% are shown. **b** Bayesian clustering for the nuclear SNP data estimated in Structure v2.3. Structure bar plot shows the genetic clusters differentiated by color. **c** Heatmap showing the *D*-statistic and its *p* value. Red color indicates higher *D*-statistics, and more saturated colors indicate greater significance

We reconstructed the phylogenetic relationships of wild and cultivated olives using nuclear, plastid, and mitochondrial SNPs separately. In addition, we tested introgression using a model-based genetic structure analysis (see “Methods”). A phylogeny based on nuclear polymorphisms (Fig. 2a) places six *sylvestris* individuals (*sylvestris*-S, W11R37, W7R224, W3R78, W2R74, W9R302) as ancestral lineages of all the other subsp. *europaea* individuals and closer to subsp. *cuspidata*. The other four *sylvestris* appear to branch within the cultivated individuals, with three of them (W1R198, W4R183, W8R225) apparently diverging after the cv. Dokkar and ancestral to the remaining cultivars. The other oleaster, *sylvestris*-T, is intermingled within the main cultivars from Lebanon and Syria. This raised the question of the authenticity or pureness of cv. Dokkar and *sylvestris*-T as cultivated or wild individuals, respectively. Genetic structure analyses including all 56 individuals suggested the presence of two distinct ancestral genetic pools, which are differentially present among the different individuals (Fig. 2b, Additional file 2: Table S5). Based on this, we distinguished three groups: one composed only by cluster 1 (four wild individuals: *sylvestris*-S, W11R37, W7R224, W3R78), another composed by cluster 2 (14 individuals: *sylvestris*-T, “Uslu,” “Barri,” “Jabali,” “Majhol-152,” “Maarri,” “Sorani,” “Beladi,” “Fishomi,” “Mari,” “Abou Satl Mohazam,” “Majhol-1013,” “Abbadi Abou Gabra-842,” “Abou Kanani”), and the largest consisting of a mixture of the two clusters in different proportions (the remaining 38 individuals). The same three genetic clusters were also supported by a principal component analysis (PCA; Additional file 1: Fig. S6). When we analyzed the oleaster samples, we observed that *sylvestris*-S, W11R37, W7R224, and W3R78 are composed only by cluster 1 (Fig. 2b), which may represent the genetic pool of western MB wild populations. The oleasters W2R74, W9R302, W1R198, W4R183, and W8R225 are composed largely by cluster 1 mixed with different amounts of cluster 2 (Additional file 2: Table S5), which could be product of gene flow between wild and cultivated populations. The oleaster *sylvestris*-T is composed only by cluster 2, similar to cultivars from Lebanon, Iran, Turkey, and Syria. Based on this and the results of the nuclear tree, we suggest that *sylvestris*-T represents a feral individual and may have been misidentified. Interestingly, all cultivars shared cluster 2, which is pervasive and enriched in cultivars from the eastern MB in different proportions, suggesting that this may be a consistent fingerprint of the primary domestication event that took place in this area [18]. The dominance of this genetic background among cultivars suggests that most cultivars mainly derive from a common primary domestication process. However, the presence of additional gene pools within the cultivars depicts patterns that could have resulted from preferential selection of genetic variants among standing variation, from separate domestication events, or from introgression events with wild populations.

To further investigate the genetic distance between populations, we calculated the fixation index (Fst) of the oleaster population composed only of cluster 1 (*sylvestris*-S, W11R37, W7R224, W3R78) and two groups of cultivars. The first group is composed only of individuals with cluster 2 (cultivars_set1, eastern cultivars), and the second is composed of individuals showing a mixture of clusters 1 and 2 (cultivars_set2, western cultivars). This analysis shows that the divergence between western oleaster and mixed cultivars (weighted Fst = 0.24) was smaller than the divergence of the western oleaster and eastern cultivars (weighted Fst = 0.41).

**Fig. 3:**
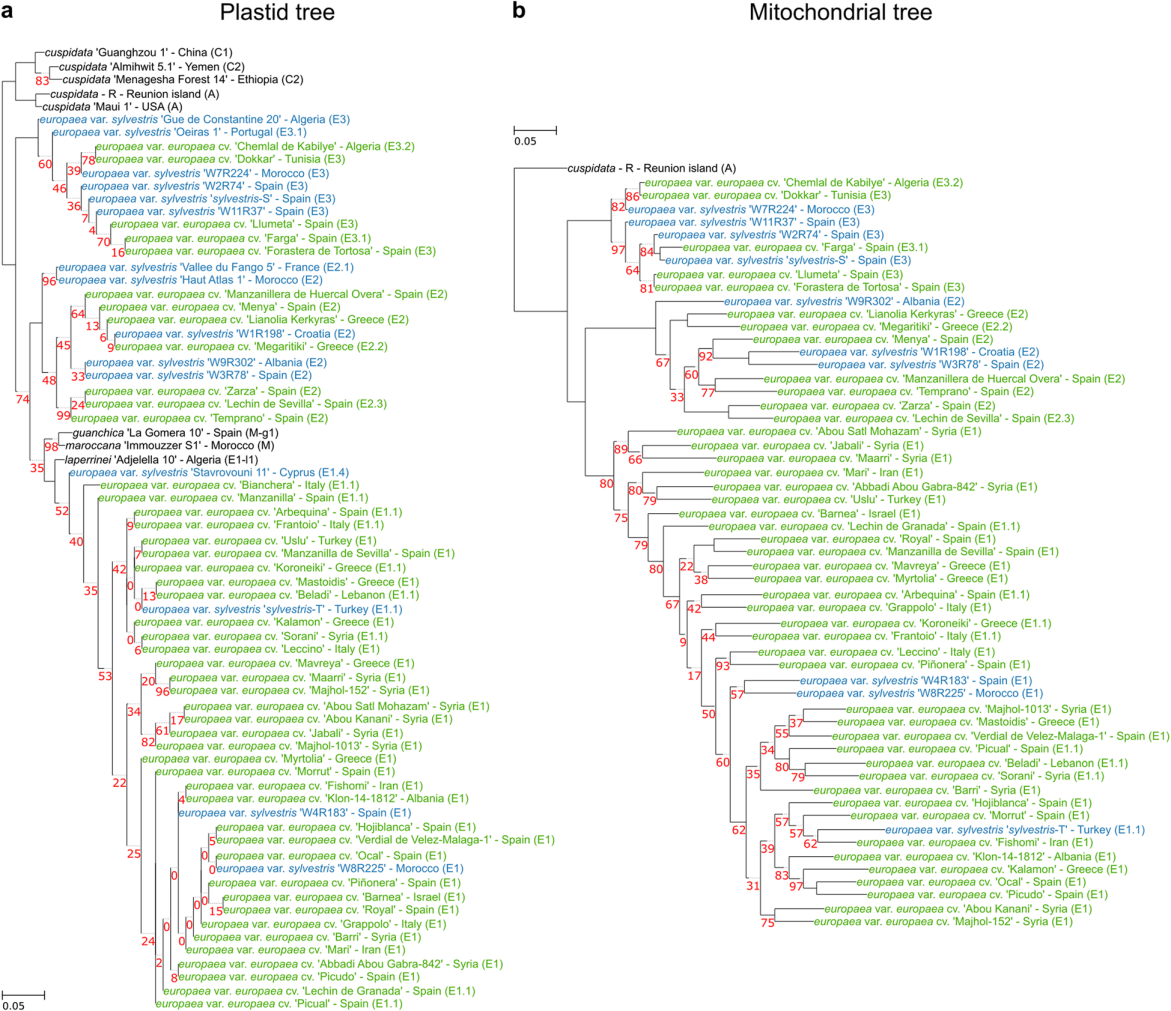
Maximum likelihood species tree derived from the organellar SNPs data. **a** Plastid phylogeny. Cultivated olives are shown in green and wild olives in blue. The geographical location of the accession and the plastid haplotype are indicated. Only bootstrap values below 100% are shown. **b** Mitochondrial phylogeny. The colors and characteristics are the same as in **a**

To reconstruct the plastid tree, we included the available plastid genomes of four additional accessions of *O. europaea* subsp. *cuspidata* and one individual of other subspecies that are publicly available (see “Methods”). In this phylogeny (Fig. 3a), *cuspidata* individuals grouped together in congruence with previous results [17, 44]. Remarkably, consistent with the plastid polymorphism patterns discussed earlier (Fig. 1b,c), we observed incongruencies between the organellar and nuclear trees (Fig. 2a and Fig. 3), which provide support for a phylogenetic signal of hybridization [46–48]. In particular, all cultivars, except the cv. Dokkar (plus the possible feral *sylvestris*-T), are monophyletic in the nuclear tree, whereas the plastid tree shows that cultivars are polyphyletic, falling into three independent clades, each of them associated with different *sylvestris* samples. Moreover, attending to the position of samples from other subspecies (such as *guanchica*, *marrocana*, and *laperrinei*), which are intermingled within the subspecies *europaea*, the plastid genome suggests a polyphyly of the subspecies *europaea*.

A particularly strong phylogenetic incongruence observed among cultivars involves the cultivars with plastid type E3 (“Farga,” “Dokkar,” “Chemlal de Kabilye,” “Llumeta,” and “Forastera de Tortosa”), which cluster together with the other cultivated olives in the nuclear tree, but are sister to *sylvestris* accessions with the same plastid type (E3), far from the other cultivars in both plastid and mitochondrial trees (Fig. 3a,b). These results suggest that the maternal line of cultivars with plastid type E3 originated from western Mediterranean wild olives (which carry the sample E3 plastid haplotype), while the paternal line originated mostly from domesticated individuals from the eastern Mediterranean basin. A similar pattern has been observed in a previous study that combined a large sample of cultivated olives and oleasters, in which most cultivars were assigned to the eastern genetic pool, even those with maternal lineages that originated from the western Mediterranean basin [11, 18]. Indeed, the largest wild populations of olives are found in the western Mediterranean. In sum, the plastid tree suggests genetic contributions, at least in the maternal lineage, of three different genetic pools that may be in agreement with multilocal introgression or domestication processes, whereas the nuclear tree suggests a dominant, congruent signal shared by all sequenced cultivars that is consistent with a unique primary domestication center in the eastern MB. These contrasting patterns reinforce the idea that cultivars are either from the eastern genetic pool or admixed forms [6, 10, 18] and support secondary admixture processes in the western Mediterranean basin, with contribution from western populations of var. *sylvestris*, as clearly shown by the plastid lineages.

### Genetic introgression from western var. *sylvestris* genetic pools among cultivated olives

**Fig. 4:**
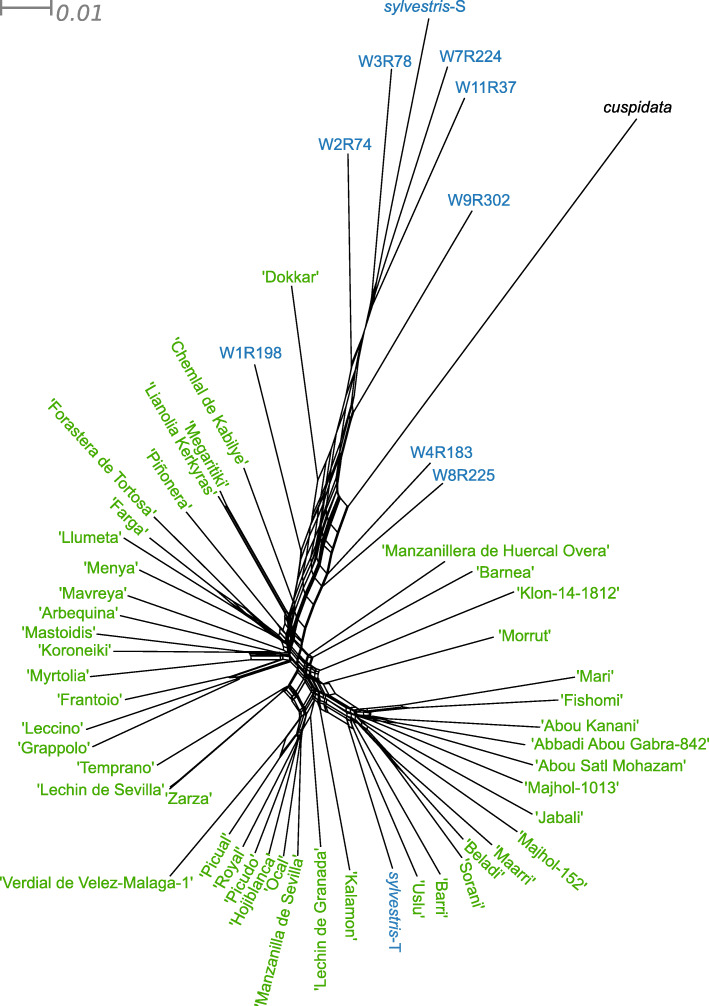
SplitsTree derived from nuclear SNPs. All the cultivars are marked in green and var. *sylvestris* in blue. The neighbor net method is used here to explore data conflict and not to estimate phylogeny

Previous phylogenetic analyses have suggested the existence of ancestral inter-subspecies hybridization in deep nodes of the evolutionary tree of *O. europaea* [26]. Our population genomics results discussed above and a recently published transcriptome-based population analysis [11] suggest recent intraspecific genetic admixture between western cultivars and western *sylvestris*. To incorporate genetic admixture into a phylogenetic framework, we reconstructed a split network tree based on nuclear genome data (Fig. 4), which revealed a heavily reticulated structure mostly affecting the relationships among all olive samples. In particular, most samples with the exception of oleasters *sylvestris*-S, W11R37, W7R224, and W3R78 appear in a heavily reticulated area. Consistent with the population genomics results, *sylvestris*-T appears well embedded within cultivars and together with other 13 cultivars show a distant connection from the other accessions. Also, cv. Dokkar and three other oleasters appear in the reticulated portion of the tree, but further apart from the other individuals.

Overall, such a reticulated network can be the result of hybridization (including introgression) or incomplete lineage sorting. In order to detect evidence of introgression among the olive samples, we ran the ABBA-BABA test [49] using the program Dsuite v0.1 r3 [50] (see “Methods”) for all quartets of the phylogenetic tree (Fig. 2a). This analysis supports the hypothesis that introgression occurred between the oleasters with a unique genetic background (Fig. 2b: *sylvestris*-S, W7R224, W11R37, and W3R78) and eight individuals (five cultivars: “Dokkar,” “Chemlal de Kabilye,” “Megaritiki,” “Lianolia Kerkyras,” “Menya,” and three oleaster: W4R183, W8R225, W1R198) with a significant *D*-statistic > 0.43 (Fig. 2c). Interestingly, the cv. Dokkar shows the highest level of introgression (*D*-statistic > 0.53), followed by the three wild individuals. These oleasters (W4R183, W8R225, W1R198) also appear closer to other cultivars in the nuclear tree and the split network tree (Fig. 2a and Fig. 4), which may point to a possible feral origin. To study whether the *D*-statistic is homogeneous or variable along the genome, we investigated the *D*-statistic over sliding windows (see “Methods”) using the five cultivars with higher signals of introgression (“Dokkar,” “Chemlal de Kabilye,” “Megaritiki,” “Lianolia Kerkyras,” “Menya”). This analysis shows that the five individuals have different amounts of introgressed genomic regions with a *D*-statistic higher than 0.5 (Additional file 1: Fig. S7a,b, Additional file 2: Table S6). Individuals with higher *D*-statistics have larger genomic regions with signatures of introgression. Then, we assessed whether specific functions were enriched in genes located within introgressed regions or non-introgressed regions (common to all five cultivars) and found no significant enrichment in either case.

In agreement with the split tree, the individuals with the genetic cluster 2 (Fig. 2b) show no signs of introgression with any of the four *sylvestris* with genetic cluster 1. This contrasts with the other analyzed cultivars that have been strongly introgressed from wild olives of the western MB (here represented by *sylvestris*-S, W7R224, W11R37, and W3R78). Importantly, the level of introgression is largely independent of the plastid haplotype (all cultivars of plastid haplotypes E2 and E3 and some of E1 show signs of introgression), suggesting that, in at least some cases, different introgressions have occurred in the maternal and paternal lineages, independently. Most of the introgressed cultivars originate from areas of the western or central MB. The 14 non-introgressed genotypes were all sampled from the eastern MB (Lebanon, Syria, Iran, and Turkey), close to the purported origin of olive primary domestication. Overall, our genome-based results are consistent with earlier results on a broader dataset and on transcriptome-based analysis [11].

### Demographic analysis suggests a population bottleneck coupled to the early domestication period

To investigate the differences in genetic diversity between cultivars and wild individuals, we used the three defined populations. The first group consisted of four oleasters with only genetic cluster 1, the second of cultivars with genetic cluster 2, and the third of cultivars with mixed genetic clusters. For the last group, we excluded cv. Dokkar for its phylogenetic position (Fig. 2b) (see “Methods”). The average pairwise nucleotide diversity based on 20-kb windows in the population of *sylvestris* (3.66 × 10^− 3^) is slightly higher than that in cultivars_set1 (3.52 × 10^− 3^) and lower than that in cultivars_set2 (4.26 × 10^− 3^). A lower nucleotide diversity in cultivars when compared with oleaster has been observed in previous studies based on inter-simple sequence repeats (ISSRs) [3], allozyme polymorphisms [51], simple sequence repeats (SSRs) [12], and plastid DNA variation [17]. Similarly, a recent transcriptome-based analysis reported slightly lower genetic diversity in cultivars compared to wild olives, leading to the suggestion of a weak-moderate population bottleneck during domestication [11].

**Fig. 5:**
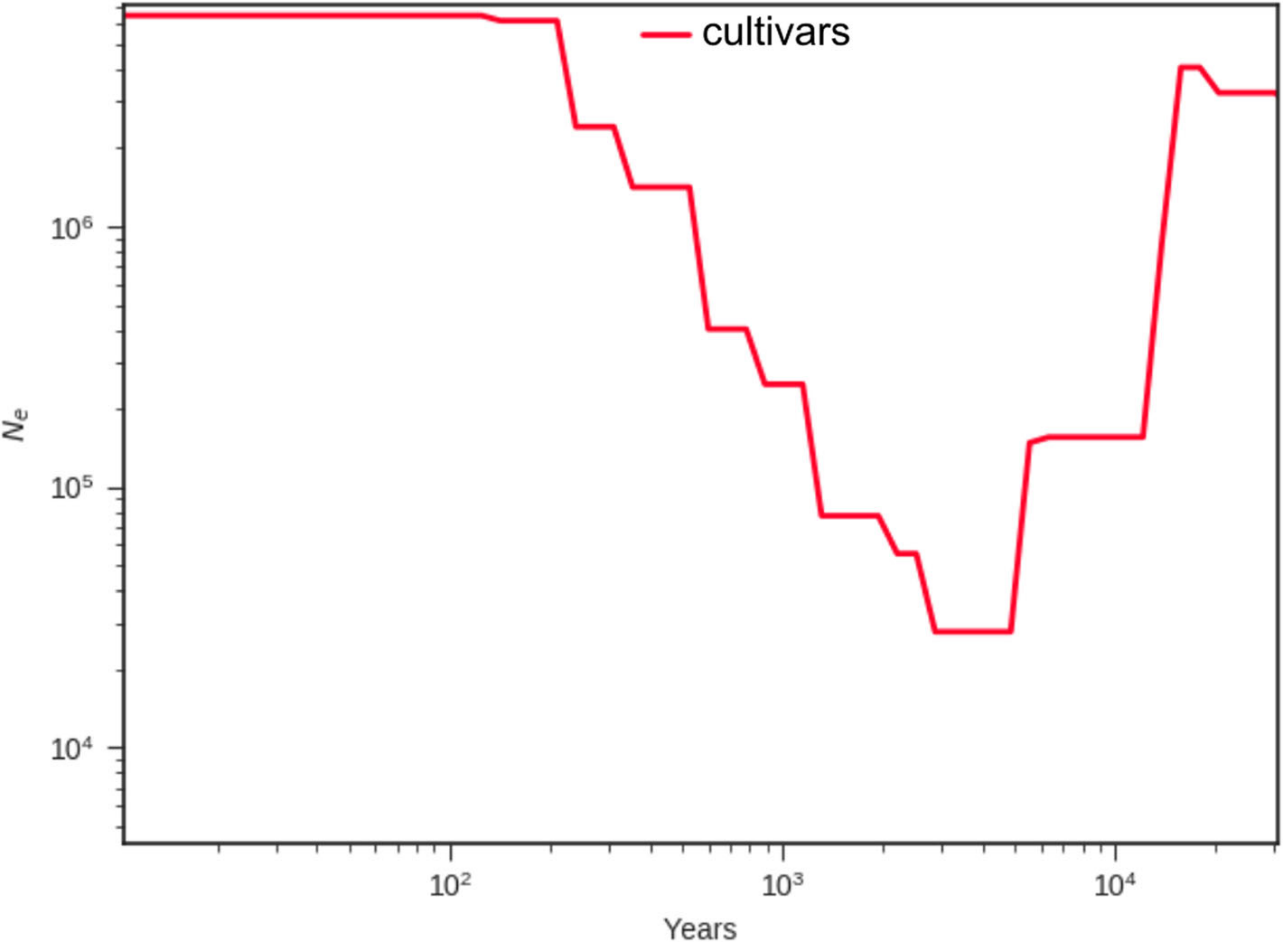
SMC++ results for inferring population size histories in cultivated olives. A generation time of 20 years was used to convert coalescent scaling to calendar time

In general, lower genetic diversity in cultivars is commonly associated with genetic bottlenecks during domestication [52]. The difference between wild and cultivated trees observed here is less pronounced than that of many domesticated plant species [52, 53]. However, perennial crops often do not show evident domestication bottlenecks, in part because vegetative propagation means that perennials may not be many generations removed from their ancestral genetic diversity [54]. In order to explore this possibility, we inferred the demographic history of olive using SMC++ v1.15.2, a tool that handles unphased genomes [55] (see “Methods”). The results of this analysis show evidence for a continuous decline in population size starting approximately ~ 14 kya, until ~ 3 kya (Fig. 5). The end of this period is close to the olive domestication timeframe (~ 6.0 kya) [8] and implies a possible domestication bottleneck. Interestingly, subsequent to olive domestication, an expansion of effective population size (Ne) can be observed. Altogether, these results suggest a mild bottleneck followed by a sustained population expansion during olive domestication.

### Identification of genes under selection

Olive trees were domesticated for their fruits, either as a source of oil or edible fruits [56]. Genes associated with agronomic traits may have undergone positive selection during domestication. In order to search for genes putatively under positive selection in the cultivars, we first classified the SNPs into intergenic, intronic, and coding. We further classified coding SNPs according to whether they imply synonymous or nonsynonymous changes (see “Methods”). Additional file 1: Fig. S8 shows that there is a higher percentage of SNPs in intergenic regions (4.8 SNPs/kb), followed by intronic (0.8 SNPs/kb) and coding regions (0.3 SNPs/kb). Moreover, the number of synonymous and nonsynonymous changes is similar across samples. In order to assess selection, we first measured the ratio of nonsynonymous and synonymous nucleotide diversity (πN/πS) in all the individuals included in this study. All had similar πN/πS values, with an average of 0.40 (Additional file 2: Table S7), suggesting similar strengths of selective pressure across all sequenced individuals. This ratio is similar to that found in other trees such as *Populus nigra* (0.48) [57] and *Populus trichocarpa* (0.40) [58].

When we analyzed the SNPs that can produce a nonsynonymous change, including heterozygous and homozygous SNPs, we found that a total of 34,060 proteins (61% of the predicted Oe9 proteome) have at least one SNP with nonsynonymous change, of which 10,385 are common for all the individuals (Additional file 2: Table S8). Proteins that did not show any nonsynonymous change might represent genes under particularly strong purifying selection and were enriched in several GO terms (see Additional file 2: Table S9).

To search for signatures of selection in olive, we decided to compare the wild population with the two different groups of cultivars (cultivars_set1 and cultivars_set2) using Tajima’s D and the McDonald-Kreitman (MK) test for all the genes with at least 4 variable sites. Tajima’s D had negative values for 13,258 genes for cultivars_set1 and 7688 for cultivars_set2, of which 4464 are common to both groups (Additional file 2: Table S10). Negative Tajima’s D values indicate an excess of low-frequency polymorphisms and can result from population expansions or strong directional selection [59]. When we tested for functional enrichment, we observed only two molecular functions enriched (structural constituent of ribosome and ADP binding) in cultivars_set1 and no enrichment for cultivars_set2. The MK test on all coding positions combined revealed a significant departure from homogeneity for both comparisons. For the first comparison, we observed that the ratio of the number of nonsynonymous to synonymous polymorphic sites within cultivars_set1 was significantly higher than that of the number of nonsynonymous to synonymous fixed sites between wild and cultivars_set1, consistent with purifying selection (Additional file 2: Table S11). For the second comparison (wild vs cultivars_set2), we observed a significant NI < 1, consistent with positive selection (Additional file 2: Table S11). When we performed the MK test in individual genes, for the cultivars_set1, we found 24 genes under positive selection and 84 genes under negative selection. For cultivars_set2, we did not find genes under positive selection and only one gene under negative selection (Additional file 2: Table S12).

The derived site frequency spectrum (SFS) of synonymous, nonsynonymous, fourfold degenerate, and deleterious SNPs was examined for the three defined olive populations (wild, cultivars_set1, cultivars_set2) using the subsp. *cuspidata* as outgroup (Additional file 1: Fig. S9). In all the cases, the SFS is left-shifted, with nonsynonymous and deleterious SNPs having higher density. When we compare the distributions of synonymous or fourfold degenerate SNPs with the nonsynonymous or deleterious SNPs, we observe slightly more differences in both groups of cultivars than in the wild population, as expected from the action of purifying selection. However, these differences only reached statistical significance in the population with more individuals (cultivars_set2) (*p* < 0.05, Mann-Whitney *U* test, see Additional file 2: Table S13).

To further determine whether one or more proteins are under positive selection in cultivated olives, we searched for evidence of recent selective sweeps by quantifying site frequency spectrum (SFS) deviations relative to genome-wide patterns using the composite likelihood ratio (CLR) statistic implemented in SweeD [60] (see “Methods”). After merging the overlapping regions (see “Methods”), we kept 258 regions for cultivars_set1 and 270 for cultivars_set2 (Additional file 2: Table S14). Shorter (< 10 kb) regions were observed in cultivars_set2 (72.2% of regions are shorter than 10 kb) than in cultivars_set1 (69.0 < 10 kb). The two groups of cultivars have two regions larger than 50 kb, but in different scaffolds. For cultivars_set1, 118 genes were identified in 94 sweep regions, and for cultivars_set2, 81 genes in 74 sweep regions, while the other regions were non-coding in both cases. Interestingly, only 4 genes are shared by both groups of cultivars. Among the genes found within sweep regions, some are associated with lipid, carbohydrate, and amino acid metabolism, and others with stress tolerance, solute transport, and RNA processing (Additional file 2: Table S15). Remarkably, we did not find any gene related to fatty acid metabolism and accumulation, although this has presumably been one of the most important characters under selection in olive domestication. Moreover, a recent study found 19 genes associated with five important agronomic traits in olive [61], and despite 14 of them being found in cv. Farga (see Additional file 2: Table S16), only five were found in the gene set with negative Tajima’s D, and none was present in the detected selective sweep regions or the genes under positive selection according to MK test. Similarly, a study based on transcriptomes of 68 different wild and cultivated samples using statistical approaches (PCAdapt [62] and BayeScan [63]) failed to identify candidate genes under selection associated with oil content or fruit size [11]. They did, however, detect ten genes as strong candidates for selection that were associated with transcriptional and translational activities and to the cell cycle. Also, it was proposed that domestication in olive may be more related to changes in gene expression than changes in protein function in agreement with its evolutionary history [11]. Further studies with more cultivated and wild samples will be needed to test this inference further.

Since some cultivars show signature of introgression with *sylvestris*-S, we assessed whether adaptive introgression has contributed to olive domestication. When we analyzed the selective sweep regions of cultivars_set1 and cultivars_set2, we found that on average 42% of these regions overlapped introgressed regions (cultivars_set1—43%, cultivars_set2—41%, see Additional file 2: Table S17), with “Chemlal de Kabilye” being the cultivar with more common regions (46%). To validate our results, per each cultivar, we randomly generated the same number of sweep regions as the ones found in cultivars_set1 and cultivars_set2, and check if the total length of overlap with introgressed regions is similar or higher than the overlap of the predicted sweep regions. In all four cultivars (“Chemlal de Kabilye,” “Megaritiki,” “Lianolia Kerkyras,” and “Menya”) analyzed, we found significant *p* values (*p* < 0.01), showing that introgressed regions contained selective sweeps more often than expected at random. The significant overlap between regions of introgression and selective sweeps suggests a role for adaptive introgression during olive domestication, as it has been observed in other crops [64].

## Discussion

### Improved assemblies for nuclear and organellar genomes

Our first reference genome assembly for *Olea europaea* (Oe6 version) [23] provided a needed resource for researchers interested in understanding the genetic basis of olive traits and the process of domestication. Here we improved the assembly (Oe9) by anchoring it to chromosomes using a publicly available genetic map [25]. In addition, we produced individual annotated assemblies for the plastid and mitochondrial genomes, which were not provided as separate assemblies in earlier releases. Our comparison of our improved nuclear assembly with the recently released assembly of an oleaster from Turkey [24] and the identification of shared duplicated regions provides additional support to the previously proposed ancient polyploidization in olives [26]. Unexpectedly, we found apparent differences in gene content even using a conservative approach that is independent of differences in annotation methodologies. These differences could reflect shortcomings of the assemblies, changes occurred during domestication, or variation in gene content among individuals, as observed in grapevine, another perennial crop [65]. Further efforts in improving reference assemblies for the olive tree with new long read technologies will definitely enhance our understanding on the effect of genome rearrangements, including deletions of genomic regions during the evolution of olive.

### Reliable genetic sources: feral individuals vs. true wild trees and cultivars

Understanding the domestication process in olives requires careful comparison between reliably identified cultivars and wild individuals. Indeed, plant choice is crucial before starting any genomic studies. In order to use reliable genetic sources, biological features of olive germplasm have to be considered in the field: (a) high phenotypic plasticity and resilience, (b) long reach of aerial transported pollen, (c) the ability to cross between genetically distinct individuals, and (d) the long lifespan of the trees (often reaching several centuries and millennia). These characteristics make it extremely difficult to tell apart pure wild individuals from those highly introgressed or even cultivars [66]. As stressed earlier, we took great care in selecting an individual (*sylvestris*-S) from an isolated wild olive population, which had been studied over the last years and meets key characteristics that conform to a true oleaster. Our phylogenetic analysis based on the nuclear genome places this wild individual among the earliest branching positions within the individuals of the subspecies *europaea*, and sister to other wild individuals, which is fully consistent with its ascription to a wild western Mediterranean genetic pool.

In stark contrast, the results for the wild sample from Turkey [24] (*sylvestris*-T) were puzzling. The phylogenetic analysis placed it at a relatively shallow clade, forming a tight cluster with thirteen cultivars from the eastern MB. This result could be explained through four hypotheses: (i) the Turkish individual identified as *sylvestris* is actually a feral olive, (ii) this tree is an oleaster highly introgressed with cultivars; (iii) considering that the cultivated olive originated from wild oleasters, this pattern may represent one of multiple primary domestication events; or, alternatively (iv) a separate, very recent domestication event. However, hypotheses (iii) and (iv) are at odds with the observed topology in which the well-defined clade formed by *sylvestris*-T and the western cultivars is placed at a shallow position of the tree, embedded within a larger clade of other cultivars, whereas sister-clade relationships to other cultivars would be expected from independent domestication events. Population structure and introgression analysis discarded hypothesis (ii), as *sylvestris*-T individual showed a genetic structure similar to the nearby sampled cultivars and no signature of introgression. In addition, the rarity of wild olive trees in the eastern Mediterranean [67] together with the small number of phenotypic characters to differentiate cultivated olives from oleaster, must be considered. The “Flora of Turkey” (page 156) states that “… spontaneous seedlings may revert to var. *sylvestris*” which, despite the misuse of the term reversion, highlights the phenotypic similarity between ferals and true oleasters in the region [67]. Earlier studies at a large geographical scale already mentioned the difficulty to find genuine oleasters in the eastern MB [51, 68]. Recent experiments confirmed this fact, analyzing putative wild olives from Turkey [15] and Israel [12] that appeared to be feral after genetic analyses. Given all these facts, we suggest that s*ylvestris-T* is a feral individual, and we have treated it as such to avoid misleading conclusions. Regarding the remaining eight *sylvestris* included, we hypothesize that only three are likely pure *sylvestris* individuals, which represent only western genetic pool, and the other five putative oleasters are likely introgressed individuals. Given the open debate on possible alternative centers of domestication, and the interest in tracing the genetic consequences of domestication in olives, the meticulous choice of additional wild populations, representing the eastern genetic pool, will be necessary. Likewise, the use of not authenticated cultivars may lead to erroneous hypotheses. Because of this reason, we excluded the putative cultivar Dokkar [16] from several analyses, given its phylogenetic position and high levels of introgression. This cultivar was not authenticated in the original collection, and its status—wild or cultivated—is controversial, according to its morphological and genetic data [9]. Thus, in order to reliably reconstruct complex domestication processes, we stress the importance of careful sampling of olive trees using ecological [69] and key morphological characters [70], assisted with molecular screening of olive material, before undertaking genome sequencing of wild and cultivated trees.

### Genomics support for a primary domestication event followed by secondary events driven by introgression from wild genetic pools

This study represents the largest phylogenetic analysis of genome-wide sequences of the Mediterranean olives. Our results, together with those from previous analyses [11, 16], suggest that cultivated individuals have similar nucleotide diversity as compared with wild individuals, being slightly higher in cultivars admixed with the western wild genetic pool, possibly due to introgression with local wild populations. Our demographic analyses support the existence of a relatively small population size at the time of domestication, with a steady decrease in population size preceding domestication as has been inferred for some other crops [71, 72]. These analyses are consistent with earlier studies suggesting a narrow distribution (and hence limited population size) of oleaster populations in the eastern MB over the last 150,000 years [8]. The demographic analysis also indicates a mild population bottleneck around 3000–14,000 years ago, consistent with the proposed period for primary domestication of olives in the Levant around 6000 years ago [8]. Interestingly, our analysis also suggests a rapid increase in population size following the domestication bottleneck, likely coupled to the expansion of olive cultures in broader Eastern Mediterranean countries. Altogether, these results suggest that one ancestral genetic pool, likely deriving from a founding population from eastern wild trees, is pervasive among cultivars. This is consistent with common ancestry at a primary domestication event from which all cultivars descend.

However, a common primary domestication event is not incompatible with subsequent nested domestication processes, perhaps driving the adaptation to local conditions or the selection of specific traits. Considering our results and previous ones from numerous molecular techniques, the emerging pattern suggests a scenario of multiple events of hybridization between individuals descending from this primary domestication event. These processes have affected cultivars from the central and western MB to different degrees. We propose that extensive gene flow between genetically rich wild and cultivated olives occurred through the expansion and diversification of olive crops by ancient Mediterranean cultures (Phoenicians, Romans, Arabs) [73]. It is likely that these introgression events, followed by artificial selection of desired characters, resulted in the incorporation of alleles from wild populations and facilitated the creation of specific olive cultivars adapted to local environments.

The origin of the introgressed genetic material can be better inferred when the donor lineage can be traced back through the maternal line. Earlier studies have shown the presence of clearly distinct haplotype groups (E1, E2, and E3) among cultivars [17, 44]. E1 is present in wild trees from the eastern MB and it is likely the signature of the primary domestication event, since it is shared by the 90% of the current olive cultivars [18]. This is particularly true for individuals sampled from regions close to the origin of domestication, cultivars from Syria, Iran, Turkey, and Lebanon and the feral individual from Turkey, which show little or no signs of introgression. Cultivars carrying the haplotypes E2 and E3, also found in wild forms of the western MB, were those often revealing a blueprint of introgression in our analysis. Particularly, the cultivars harboring the E3 plastid haplotype have a different phylogenetic history than that of the other cultivars suggesting secondary hybridization of cultivars with wild oleasters from the Iberian Peninsula, in which a lineage similar to var. *sylvestris* from Spain or Morocco acted as the maternal line. Consistent with this, the nuclear genome of these cultivars shows signs of introgression with var. *sylvestris* from western MB. However, this introgression signal is also detected in other cultivars, irrespective of the plastid haplotype lineage. Altogether, these results suggest that admixture with wild individuals from western populations of var. *sylvestris* has been common and has taken place multiple times, both in the maternal and paternal lineages.

Based on plastid haplotypes of cultivars, introgression from at least two different wild genetic pools, other than the one involved in the primary domestication, has contributed to the olive germplasm. These different genetic pools are highly divergent from each other, as shown in our phylogenetic reconstructions from plastid genomes, which also suggest that the subspecies *europaea* might have multiple origins (polyphyletic). Some other studies with plastid and nuclear markers have shown similar results [2, 3, 44]. Despite the detectable signal of introgression in nuclear genomes, all cultivars, including introgressed ones, are monophyletic in our reconstructions (considering *sylvestris*-T as a feral). This is also the case of the cv. Farga, which shares a maternal ancestor with the *sylvestris-S* individual analyzed here. These two individuals are closely related when organellar genomes are used for phylogenetic reconstruction, but cv. Farga appears well embedded within the cultivar clade in the nuclear genome phylogeny. This indicates that the introgressed material in the nuclear genome has been reduced through subsequent crosses following the initial hybridization. Altogether, and in contrast to a recent study focused on olive domestication [16], the phylogenetic and admixture analyses show that rampant hybridization has shaped the evolutionary history of the different lineages of olives. We thus hypothesize that olives represent a domestication continuum [6]. Following a primary domestication event in the eastern MB, there were additional hybridizations with local wild genetic pools throughout the MB. Although there may be some evidence and rationale for calling these hybridizations secondary domestication events [12], all of the germplasm in our analyses contain some remnant of the primary event. We note, however, increased genomic sampling, particularly of truly wild populations from the Levant, is needed to help describe these complex patterns of evolution in *Olea europaea* across the numerous areas of the Mediterranean basin.

### Lack of a clear domestication syndrome

Cultivated olives have undergone a complex domestication process, which has led to morphological and physiological changes. The main observable traits selected during the transition between oleasters and cultivars are fruit size and oil content [13, 56, 70]. The domestication scenario described in the previous section, which is punctuated by hybridization, may make it difficult to detect genes selected during the process of domestication. We used three different approaches (McDonald-Kreitman test, Tajima’s D, and selective sweeps prediction) to detect sequence changes likely associated with selection. We detected different sets of genes putatively selected for cultivars_set1 and cultivars_set2 with few genes overlapping the different methods. In general, genes positively selected in cultivated olives were associated with a response to biotic and abiotic stresses. Interestingly, five genes detected with negative Tajima’s D are related to genes recently described as important for fruit weight and stone weight [61]. However, further analyses are needed to ascertain whether they indeed play a role in trait selection during domestication (see [74] for secondary compounds). Previous analyses detected few signs of positive selection affecting protein-coding regions, but they did detect differences in expression levels between cultivars and wild individuals for specific genes [11]. This led to the conclusion that selection may have acted on non-coding regions that drive gene expression. However, given the difficulty of controlling for other factors driving gene expression (different periods of the year, local environmental conditions), we believe this result must be viewed with caution. Also, a recent study showed that the activation of transposable elements near genes of important agronomic traits may play a role in olive domestication [16]. Alternative approaches are needed to detect alleles whose presence in the cultivars were selected through domestication. We cannot rely on models that assume only vertical evolution, but would rather search for shared conserved or introgressed regions across different cultivars sharing similar phenotypes [70]. To be effective, a much larger sampling of genomic sequence will be needed for such an approach. In addition, assemblies from representative lineages of reliable wild olives will help to better trace the origin of different introgressed regions in the genomes of cultivars. Overall, our new phylogenomic and genetic analyses of whole genome sequences show evidence for a complex process of reticulation disrupting historical isolation in the course of olive domestication.

## Conclusions

The comparison of the improved reference genome of *Olea europaea* cv. “Farga” (version Oe9) with the other two available genomes and all sequenced individuals showed a different gene content among individuals.

Phylogenetic and introgression analysis helped to uncover the feral origin of six samples. Remarkably, our results suggest that the available genome of var. *sylvestris* corresponds to a feral individual.

Genes positively selected in cultivated olives were associated mainly with response to biotic and abiotic stresses. We did not find evidence of selection of genes associated with fruit size and oil content.

Rampant hybridization has shaped the evolutionary history of the different lineages of olives. After the primary domestication in the eastern Mediterranean basin, there were numerous secondary events across most countries of southern Europe and northern Africa, often involving genetic admixture with genetically rich wild populations, particularly from the western Mediterranean Basin.

## Methods

### Scaffolding of the cultivated olive genome using a linkage map

A new, improved version of the *O. europaea* genome assembly (Oe9) was produced by anchoring the Oe6 version [23] to a publicly available genetic map [25] using ALLMAPS [75]. First, we took the intersection of 7042 markers for which a sequence was provided and mapped them with BWA v0.7.15 [76] to the Oe6 olive genome assembly. Filtering for minimum mapping quality 20 and fewer than 10 mismatches, we obtained 5780 mappings. Intersecting these mapped markers with the 3404 markers placed in the genetic map resulted in a set of 2759 markers with both a genetic map position and an unambiguous physical location in the Oe6 assembly. ALLMAPS was then run with default parameters. A total of 2362 markers were considered unique, of which 2134 were anchored and 228 unplaced.

### Functional annotation of the cultivated olive genome

In order to give more functional insight to our analysis, we decided to improve the functional annotation of the genes present in the Oe6 version of the genome by running Blast2go [77], which in turn ran a BLASTP [78] search against the non-redundant database (April 2018) and Interproscan [79] to detect protein domains on the annotated proteins.

### Comparison of the *europaea* and *sylvestris* genomes

To compare the gene completeness of the four available genome assemblies of *O. europaea* subsp. *europaea* (two var. *europaea* of cv. Farga, one cv. Picual, and one var. *sylvestris*), the BUSCOv3 (Benchmarking Universal Single-Copy Orthologs) program was run using the embryophita_odb9 database made of 1440 conserved orthologous genes in plants (Additional file 2: Table S1). To better characterize gene completeness differences between the cvs. Farga and Picual genomes assemblies that had similar BUSCO results, two RNAseq datasets obtained from immature cv. Farga olives (ERR1406351) and cv. Arbequina seeds (SRR6003535) were aligned to the two assemblies with STAR. Total percentage of reads mapped, as well as percentage of unique mappings, has been reported (Additional file 2: Table S2).

Additionally, to compare the three available genome assemblies of var. *europaea* (“Farga” v. Oe6 and Oe9, “Picual”) and *sylvestris*, we plotted their cumulative assembly lengths using the python package seaborn [80]. The predicted protein-coding gene sequences of the two assemblies were compared using a search with BLASTN [27]. Results were filtered using cutoffs of 80% identity and e-value < 1e−5. For the genes that did not have a hit, we analyzed whether they were covered by reads of the other sequenced accession. For this step, we first mapped the reads of each genome against the other using BWA 0.7.6a-r433 [76]. We considered a gene as individual-specific (e.g., *europaea*-specific) if it did not pass a filter of coverage > 20 over 50% of the gene length in the other genome (e.g., *sylvestris*). With the aim of achieving a more conservative set of individual-specific genes, we applied a more stringent filter of coverage (> 5). For the list of detected individual-specific genes found in *europaea*, we mapped the reads of all the varieties and an outgroup (subsp. *cuspidata)* using the same approach. Bedtools v2.26.0 was then used to detect coverage and a gene was assumed to be present in a variety when at least 90% of the coding region was mapped by reads (coverage filter > 5). The set of genes with no reads mapping from any other variety was scanned for additional contamination. In total, 104 genes were discarded due to the fact that they did only have hits in other non-plant species.

Finally, in order to search for the functions of the genes without a hit in the other genome (individual-specific), we performed a BLAST search against the NCBI non-redundant database, and the same cutoffs as described before.

### Genome sequences

We sampled and sequenced twelve genomes of *O. europaea*: ten cultivars (“Arbequina”, “Beladi”, “Picual,” “Sorani,” “Chemlal de Kabilye,” “Megaritiki,” “Lechin de Sevilla,” “Lechin de Granada,” “Frantoio,” and “Koroneiki”), one oleaster (*sylvestris*-S), and one subsp. *cuspidata* to be used as an outgroup. The inclusion of outgroups in phylogenomic analyses is recommended as they help to provide a temporal polarity to the data (e.g., distinguish between ancestral and derived characters). These samples broadly covered the geographical distribution of the species in the MB (see Table 1). The authenticity of these cultivars was previously substantiated through molecular and morphological markers [9]. The sequenced oleaster (referred to as *sylvestris*-S from now onwards) was collected in the North of Spain. All the samples were collected according to the local, national, or international guidelines and legislation.

The DNA of all individuals was extracted as described in [23], and their genomes were sequenced using Illumina HiSeq 2000 paired-end technology to a sequencing depth ranging from 24 to 34× at the CNAG-CRG sequencing facilities, as described for the reference genome [23]. In addition to these individuals, we used publicly available data of the reference genome sequence of the olive cv. Farga [23]. “Farga” is a cultivar from Catalonia (eastern Spain) with the E3.1 plastid haplotype and previously classified as representative of the central MB cultivated genepool (Table 1). We also used a published assembly of *O. europaea* var. *sylvestris* (referred as *sylvestris*-T from now onwards) [24], and 49 samples from a recent publication [16], and downloaded fourteen plastid genomes of the Mediterranean olive from the NCBI database (see Table 1).

### Organelle assemblies

The available reference genome sequence does not include separate scaffolds for mitochondrial and plastid genomes [23]. Here, we assembled and annotated both organellar genomes of the cv. Farga using paired-end (PE) and mate-pair (MP) data from the reference genome sequence project [23]. Briefly, all genome shotgun Illumina reads were mapped using BWA v0.7.13-r1126 [76] to a reference plastid sequence (NC_013707), and a mitochondrial sequence (MG372119). Then reads were filtered allowing only those that mapped in proper pairs with a hard and soft clipping for a maximum of the 25% of the total length of the read. The plastid genome was assembled using NOVOPlasty v2.6.3 [81]. The mitochondrial genome was first assembled with SPAdes v3.10.0 [82]. Then, this initial assembly was scaffolded using SSPACE_Basic v2.0 [83] using PE and MP libraries. Finally, gaps were filled with GapFiller v1–10 [84].

The plastid and mitochondrial genomes were annotated by BLAST searches against previously annotated plastid (FN996972, MG255763) and mitochondrial (MG372119, KX545367) genomes. Gene structures (i.e., intron–exon boundaries) were defined using Exonerate v2.47.3 [85] using the “protein2genome” model. Annotations of tRNA genes were verified using tRNAscan-SE [86].

### Detection of single nucleotide variants

To assess nucleotide diversity across sequences of the Mediterranean olive at the nuclear, plastid, and mitochondrial levels, we called single-nucleotide polymorphisms (SNPs) using the cv. Farga genome as a reference for all the cases (for the nuclear genome we used the Oe9 version). Sequenced reads from each individual were mapped against the reference genome using BWA 0.7.6a-r433 [76], and SNPs were identified with GATK HaplotypeCaller v4.0.8.1 [87], setting ploidy to 2, and using thresholds for mapping quality (MQ > 40), quality by depth (QD > 2), Fisher strand bias (FS < 60), mapping quality rank sum test (MQRankSum > − 12.5), read pos rank sum test (ReadPosRankSum > − 8), strand odds ratio (SOR < 3), read depth of coverage (DP ≥ 10) in at least the 80% of individuals, and allelic depth (AD ≥ 5) in at least the 80% of individuals. Sites with missing alleles and spanning a deletion were also removed. Finally, VCFtools v0.1.17 [88] was used to filter out positions according to the number of alleles (--max-alleles 2) and minor allele frequency (--maf 0.008).

### Admixture mapping

We used Bayesian hierarchical clustering and principal component analysis (PCA) of genetic variance to identify population structure without a priori grouping assumptions among individuals of the subsp. *europaea*. For the Bayesian hierarchical clustering, we used the Structure software v2.3.4 (89) and because of the large number of polymorphic positions in the nuclear genomes of the *O. europaea* samples, and computational limitations, we generated ten subsets of 100,000 randomly chosen polymorphic positions without overlaps and analyzed them in parallel. Structure was run with 100,000 generations of “burn-in” and 100,000 Markov chain Monte Carlo (MCMC) iterations after burn-in for increasing *K* values ranging from 1 to 6, considering independent alleles and admixture of individuals. Simulations were repeated five times for each value of *K*. The optimal number of genetic clusters was determined using the Δ*K* method [89] using the software Structure Harvester [90]. The optimal *K* value was visualized with DISTRUCT v1.1 [91]. For PCA analyses, we used PLINK v2.00a2.3LM [92] and the complete set of SNPs (24,724,756 positions). Finally, population differentiation (weighted FST) was calculated using VCFtools v0.1.17 [88].

### Phylogenetic analysis

Phylogenetic trees were reconstructed using SNP data from nuclear, plastid, and mitochondrial genomes separately. In each case, the genome sequence of each sequenced individual was obtained by replacing the SNP positions in the respective reference genome, resulting in a pseudo-alignment of all the sequenced genomes. Specifically, for the nuclear dataset, we included only homozygous SNPs. For the plastid genomes, we included additional sequences by aligning our genomes with the database genomes (see Table 1) using MAFFT v7.305b [93]. All these alignments were trimmed using trimAl v1.4 [94] with the options -st 1 and -complementary, in order to remove all the non-informative positions. The final alignment had 12,862,844 variable positions for the nuclear genome, 327 for the plastid genome, and 4168 for the mitochondrial genome. Phylogenetic trees were reconstructed from these alignments using RAxML v8.1.17 [95] and the GTR model as it is the most frequent evolutionary model found in previous studies. Support values were calculated based on 100 bootstrap searches using the rapid bootstrapping as implemented in RAxML. Additionally, for the nuclear data, we reconstructed a phylogenetic network using SplitsTree4 v4.14.5 and the NeighborNet approach [96]. Since we had six cultivars (“Koroneiki,” “Lechin de Sevilla,” “Arbequina,” “Chemlal de Kabylie,” “Picual,” “Frantoio”) that had two samples and in the nuclear tree cluster together, we decided to only keep the individuals that were sequenced in this project. After this filter, we keep 46 cultivars, 10 *sylvestris*, and 1 subsp. *cuspidata*, which were used for all the analysis.

### Analysis of introgression with SNP data

The ABBA-BABA test [49] was used to search for evidence of introgression among the olive samples. Dsuite v0.1 r3 [50] was employed to calculate the *D*-statistic from nuclear SNP data for all subsets of quartets that were compatible with the previously reconstructed phylogenetic tree (see above). Each quartet includes the tree subsp. *europaea* individuals plus the subsp. *cuspidata* as outgroup (Additional file 2: Table S18). For multiple hypotheses testing, we applied a false discovery rate correction to the *p* values [97]. Then a heatmap showing the *D*-statistic and its *p* value was plotted for all pairs of individuals using the plot_d.rb script (https://github.com/mmatschiner/tutorials/blob/master/analysis_of_introgression_with_snp_data/src/plot_d.rb). The Dsuite Dinvestigate tool was used in sliding windows of 5000 SNPs, incremented by 1000 SNPs to test whether the *D*-statistic is homogeneous or variable throughout the genome. It was tested in the trios with the strongest signals of introgression: “Beladi”-“Dokkar”-*sylvestris*-S, “Beladi”-“Chemlal de Kabilye”-*sylvestris*-S, “Beladi”-”Megaritiki”-*sylvestris*-S, “Beladi”-”Menya”-*sylvestris*-S, “Beladi”-”Lianolia Kerkyras”-*sylvestris*-S. Then, the overlapping regions with *D*-statistic > 0.5 were joined per each of the five cultivars analyzed and genes present in these regions were used for enrichment analysis.

### Nucleotide diversity

Pairwise nucleotide diversity (*π*) was calculated using VCFtools v0.1.17 [88] per window of 20 kb. The samples were grouped together into three datasets: oleaster (only the *sylvestris* with 100% of cluster 1 as predicted with Structure (Additional file 2: Table S5)), cultivars_set1 (cultivars with genetic cluster 2), cultivars_set2 (cultivars with clusters 1 and 2). The *sylvestris* with mixed clusters (W2R74, W9R302, W1R198, W4R183, W8R225) and *sylvestris*-T were excluded because of their possible feral origin. The cv. Dokkar was also excluded for its phylogenetic position and high levels of introgression (see Fig. 2a,b).

### Demographic history of cultivated olives

To estimate population size histories in olive, we employed SMC++ v1.15.2 [55], which is capable of analyzing unphased genomes. The dataset included only 45 cultivars, we decided to exclude “Dokkar” for its phylogenetic positions and high levels of introgression (see Fig. 2a,b). First, we masked all regions larger than 5 kb with a coverage < 10× in at least one of the samples included using bedtools v2.26.0 [98]. Then, we ran SMC++ using default parameters and setting the T1, the most recent time point for population size history inference, to 10. Finally, a generation time of 20 years [12] and a mutation rate of 7.77 × 10^− 9^ mutations per nucleotide per generation [99] were used to convert the scaled times and population sizes into real times and sizes.

### SNP characterization

Nuclear SNPs were classified according to their genomic location as intergenic, intronic, and coding. Coding SNPs were further classified into synonymous and nonsynonymous, according to the implied change in the respective codon. We also differentiate the fourfold degenerate positions and deleterious SNPs (if a nonsynonymous SNP produces a change in the start or stop codon). For the heterozygous positions, if at least one of the alleles was nonsynonymous, we classified the position as nonsynonymous. GO term enrichment analyses of the proteins without nonsynonymous SNPs was calculated using FatiGO [100]. To investigate the variation of nonsynonymous and synonymous SNPs within coding regions, we compared nonsynonymous changes per nonsynonymous site (πN) to synonymous changes per synonymous site (πS) using a synonymous: nonsynonymous site ratio of 1:3 [101].

### Identification of genes under selection

To detect protein-coding genes that have potentially undergone selection among the cultivated individuals, we used different approaches. Tajima’s D neutrality test was performed on coding sequences of all genes with at least 4 SNPs using VCFtools v0.1.17 [88] for each defined olive population (see section “Nucleotide diversity”). To evaluate if the ratio of synonymous to nonsynonymous polymorphisms within individuals of cultivars (cultivars_set1 and cultivars_set2 independently) will be similar to the ratio of synonymous to nonsynonymous divergences between cultivars and wild populations (fixed differences), we performed a McDonald-Kreitman test for all coding regions [102]. We also reported the neutrality index (NI), which shows the directionality of the McDonald-Kreitman test, and the *p* value was calculated using Fisher’s exact test, which evaluates if the differences of the ratios are significant. An NI value > 1 is consistent with negative selection, while an NI value < 1 is consistent with positive selection. For multiple hypotheses testing, we applied a false discovery rate correction to the *p* values [97]. However, after the correction, none of the genes shows significant *p* values, but anyway we decided to show the few genes with *p* < 0.05. We also generated the derived site frequency spectrum (SFS) for synonymous, nonsynonymous, fourfold degenerate, and deleterious sites using subsp. *cuspidata* to determine ancestral state. Then we compared the SFS distribution of probable neutral evolving sites (synonymous and fourfold degenerate) with no neutral evolving sites (nonsynonymous sites and deleterious) using the Mann-Whitney *U* test. Additionally, an approach based on the site frequency spectrum (SFS). SweeD v3.1 [60] was used to identify selective sweeps in olive. This program is based on Sweepfinder [103] and uses a composite likelihood ratio (CLR) to identify loci showing a strong deviation in the site frequency spectrum (SFS) toward rare variants. We used the subsp. *cuspidata* as an outgroup to infer ancestral alleles. SweeD was run separately for each scaffold and grid as the only parameter. The grid parameter was calculated per scaffold in order to have a measure of the CLR every 5 kb (size of the scaffold/5000). Outliers were defined as the 0.5% with the most extreme *p* values. Closer regions, with less than 10 bp distance, were collapsed. Finally, for all the protein lists generated, we performed a GO term enrichment analysis using FatiGO [100].

### Identification of overlapping sweep and introgressed regions

In order to test whether adaptive introgression played an important role in olive domestication, we used the sweep regions generated for cultivars_set1 and cultivars_set2 (Additional file 2: Table S14) and compared with the predicted introgressed regions for cultivars “Chemlal de Kabilye”, “Megaritiki”, “Lianolia Kerkyras” and “Menya” (Additional file 2: Table S6). From the sweep regions, only those with 100% overlap were selected. To validate our results, we calculated an empirical *p* value for each cultivar that tests whether random generated regions have larger overlap with the introgressed regions than the predicted sweep regions. Briefly, the same number of sweep regions were randomly generated with similar sizes, and then we check if the total length of overlap with introgressed regions (sampled) is the same or larger than that of the sweep regions (observed). We repeated this procedure 1000 times and calculated the *p* value by dividing the number of times where the length of sampled regions ≥ observed regions by the number of repetitions.

## Supplementary information

**Additional file 1 Fig. S1.** Genome comparison of *sylvestris* and *europaea.* (a) Cumulative genome length per scaffold ranked in order of size for the genome assembly of *europaea* (Oe9 - green, Oe6 - olive, picual - orange) and *sylvestris* (blue). A straight vertical line represents a perfect genome assembly. The horizontal plateaus indicate many small scaffolds. The top right end of each curve shows the total number of scaffolds. (b) Syntenic plot of the genome of *europaea* against *sylvestris* generated by SynMap. (c) Histogram of log 10 transformed Ks values of syntenic gene pairs identified as calculated by SynMap. **Fig. S2.** Plastid genome of the cultivar Farga. Protein coding genes are shown in green, rRNAs in light blue, and tRNAs in purple. The SNPs are shown per each individual sequenced in this study in the following order starting from outside: ‘Arbequina’, ‘Picual’, ‘Beladi’, ‘Sorani’, ‘Koroneiki’, ‘Frantoio’, ‘Lechin de Granada’, ‘Lechin de Sevilla’, ‘Megaritiki’, ‘Chemlal de Kabilye’, *sylvestris*-T, *sylvestris*-S. **Fig. S3.** Mitochondrial genome of the cultivar Farga. Protein coding genes are shown in green, rRNAs in light blue, and tRNAs in purple. The SNPs are shown per each individual sequenced in this study in the following order starting from outside: ‘Arbequina’, ‘Picual’, ‘Beladi’, ‘Sorani’, ‘Koroneiki’, ‘Frantoio’, ‘Lechin de Granada’, ‘Lechin de Sevilla’, ‘Megaritiki’, ‘Chemlal de Kabilye’, *sylvestris*-T, *sylvestris*-S. **Fig. S4.** Homozygous SNP distribution along the nuclear genome. The SNPs are shown in windows of 100 Kb. Cultivars are plotted in green and sylvestris in blue. Since cv. Farga was used as a reference genome, we do not expect homozygous SNPs for this sample. **Fig. S5.** Heterozygous SNP distribution along the nuclear genome. The SNPs are shown in windows of 100 Kb. Cultivars are plotted in green and sylvestris in blue. **Fig. S6.** Principal component analysis (PCA) based on 24,724,756 nuclear SNPs. The first two principal components (PC1 and PC2) are plotted, which explained 35.17% and 11% of the total variance, respectively. The individuals are shown with three different colors according to the results of structure (Fig. 2b): blue for individuals composed mainly of cluster 1, green for individuals composed mainly of cluster 2, and orange for mixed individuals. The cultivars are identified by a circle and the oleaster by a cross (x). **Fig. S7.** Patterns of introgression. (a) D statistics across the largest scaffold (Oe9_LG02) in windows of 5Kb and step size of 1Kb for the analysis of the trios: ‘Beladi’-‘Dokkar’-*sylvestris*-S, ‘Beladi’-‘Chemlal de Kabilye’-*sylvestris*-S’, ‘Beladi’-‘Megaritiki’-*sylvestris*-S, ‘Beladi’-‘Menya’-*sylvestris*-S, ‘Beladi’-‘Lianolia Kerkyras’-*sylvestris*-S. The horizontal red line marks D = 0.5. (b) Genomic regions introgressed with a D > 0.5 in ‘Megaritiki’ (grey), ‘Menya’ (blue), ‘Lianolia Kerkyras’ (orange), ‘Dokkar’ (green), and ‘Chemlal de Kabilye’ (red). Percentages below the cultivar names represent the percentage of the genomic region that is introgressed in the cultivar. **Fig. S8.** Number of homozygous and heterozygous SNPs (SNPs/Kb) in the intergenic, intronic and coding region of the genome. The coding region was divided according to the changes that the allele can produce (synonymous and nonsynonymous). **Fig. S9.** Derived site frequency spectrum (SFS) of synonymous, four fold degenerate, non-synonymous, and deleterious SNPs. (a) Wild populations, (b) Cultivars_set1, (c) Cultivars_set2. Subspecies *cuspidata* was used as an outgroup for identifying the derived allele.

**Additional file 2 Table S1.** General characteristics of the four genome assemblies of the cv. Farga (Oe6, Oe9), picual, and the genome assembly of the var. *sylvestris* from Turkey. **Table S2.** Total percentage of reads mapped from two RNAseq libraries, as well as, percentage of unique mappings to the Oe9 and picual genomes. **Table S3.** List of unique genes of *europaea* and *sylvestris* with their homologous function. **Table S4.** General characteristics of the plastid and mitochondrial genomes of the cultivar Farga. **Table S5.** Admixture coefficient (Q) of each individual per cluster. This table was used to create the Fig. 2b. **Table S6.** Introgressed regions per each cultivar. Columns in order show: cultivar name (‘Chemlal de Kabilye’, ‘Megaritiki’, ‘Lianolia Kerkyras’, ‘Menya’, and ‘Dokkar’), scaffold, start of the region, end of the region, D-statistic, genes present in the region. **Table S7.** Number of synonymous, nonsynonymous, four fold degenerate, affecting stop codons, affecting start codons SNPs for homozygous and heterozygous positions per individual. TheπN/πS ratio of homozygous SNPs, πN/πS ratio of heterozygous SNPs, and πN/πS ratio of total number of SNPs is also shown. **Table S8.** Number of proteins with SNPs and with nonsynonymous SNPs per individual. **Table S9.** GO terms enriched in the list of proteins that do not have a nonsynonymous SNP. First column shows the term category, the second, the GO term, the third, the term level, the fourth, the *p*-value, and the fifth, the term name. **Table S10.** List of genes with negative Tajima’s D. The columns in order indicate: the group of cultivars (cultivars_set1 or cultivars_set2), gene, scaffold, number of SNPs, Tajima’s D. **Table S11.** Result of McDonald and Kreitman test for all coding regions of wild vs cultivars_set1 and wild vs cultivars_set2. **Table S12.** Result of McDonald and Kreitman test for each gene. The columns in order show: the comparison for the test (wild vs cultivars_set1 or wild vs cultivars_set2), number of fixed nonsynonymous sites (Fn), number of fixed synonymous sites (Fs), number of polymorphic nonsynonymous sites (Pn), number of polymorphic synonymous sites (Ps), the ratio of fixed nonsynonymous sites per synonymous sites (Fn/Fs), the ratio of polymorphic nonsynonymous sites per synonymous sites (Pn/Ps), the neutrality index (NI), p-value (Fisher’s exact tests), homologous function. **Table S13.** Derived site frequency of synonymous, nonsynonymous, deleterious, and four fold degenerate sites. The *p*-values using the Mann-Whitney U test of the comparison of neutral and no neutral sites are also shown. **Table S14.** Selective sweeps for cultivars_set1 and cultivars_set2. The genes that are present in the regions are indicated. **Table S15.** List of proteins in regions with selected sweeps and their associated function. **Table S16.** Blast results of the 19 genes of *sylvestris*-T against cv. Farga. The results were filtered by %identity > 90 and e-value<1e-5. Asterisk mark genes with negative values of Tajima’s D in cultivars_set1 (*), in cultivars_set2 (**), and in both sets of cultivars (***). **Table S17.** Selective sweeps present in introgressed regions of ‘Chemlal de Kabilye’, ‘Megaritiki’, ‘Lianolia Kerkyras’, and ‘Menya’. **Table S18.** D-statistic of all trios of subsp. *europaea* analysed. The columns show the name of the individual 1, individual 2, individual 3, D-statistic, p-value, and adjusted p-value.

## Data Availability

All data generated or analyzed during this study are included in this published article, its supplementary information files and publicly available repositories. The new version of the *O. europaea* var. *europaea* genome and the resequencing data produced in this project have been deposited in the ENA (European Nucleotide Archive) under the accession number PRJEB35540. The genome assembly and annotation are also available in https://denovo.cnag.cat/olive, together with a JBrowse genome browser and a Blast Server.

## References

[1] Green PS (2002). A revision of olea L. (oleaceae). Kew Bull.

[2] Besnard G, Rubio de Casas R, Christin P-A, Vargas P (2009). Phylogenetics of Olea (Oleaceae) based on plastid and nuclear ribosomal DNA sequences: tertiary climatic shifts and lineage differentiation times. Ann Bot.

[3] Vargas P, Kadereit JW (2001). Molecular fingerprinting evidence (ISSR, Inter-Simple Sequence Repeats) for a wild status of *Olea europaea* L. (Oleaceae) in the Eurosiberian North of the Iberian Peninsula. Flora..

[4] Rubio de Casas R, Besnard G, Schönswetter P, Balaguer L, Vargas P (2006). Extensive gene flow blurs phylogeographic but not phylogenetic signal in Olea europaea L. Theor Appl Genet.

[5] Zohary D, Spiegel-Roy P (1975). Beginnings of fruit growing in the old world. Science..

[6] Kaniewski D, Van Campo E, Boiy T, Terral J-F, Khadari B, Besnard G (2012). Primary domestication and early uses of the emblematic olive tree: palaeobotanical, historical and molecular evidence from the Middle East. Biol Rev Camb Philos Soc.

[7] Terral J-F, Alonso N, RB i C, Chatti N, Fabre L, Fiorentino G (2004). Historical biogeography of olive domestication (Olea europaea L.) as revealed by geometrical morphometry applied to biological and archaeological material. J Biogeogr.

[8] Besnard G, Khadari B, Navascués M, Fernández-Mazuecos M, El Bakkali A, Arrigo N (2013). The complex history of the olive tree: from Late Quaternary diversification of Mediterranean lineages to primary domestication in the northern Levant. Proc Biol Sci.

[9] Trujillo I, Ojeda MA, Urdiroz NM, Potter D, Barranco D, Rallo L (2014). Identification of the Worldwide Olive Germplasm Bank of Córdoba (Spain) using SSR and morphological markers. Tree Genet Genomes.

[10] Besnard G, Rubio de Casas R (2016). Single vs multiple independent olive domestications: the jury is (still) out. New Phytol.

[11] Gros-Balthazard M, Besnard G, Sarah G, Holtz Y, Leclercq J, Santoni S, et al. Evolutionary transcriptomics reveals the origins of olives and the genomic changes associated with their domestication. Plant J. 2019; 10.1111/tpj.14435. 10.1111/tpj.14435 PMC6851578 31192486

[12] Diez CM, Trujillo I, Martinez-Urdiroz N, Barranco D, Rallo L, Marfil P (2015). Olive domestication and diversification in the Mediterranean Basin. New Phytol.

[13] Breton C, Terral J-F, Pinatel C, Médail F, Bonhomme F, Bervillé A (2009). The origins of the domestication of the olive tree. C R Biol.

[14] Díez CM, Gaut BS (2016). The jury may be out, but it is important that it deliberates: a response to Besnard and Rubio de Casas about olive domestication. New Phytol.

[15] Yoruk B, Taskin V (2014). Genetic diversity and relationships of wild and cultivated olives in Turkey. Plant Syst Evol.

[16] Jiménez-Ruiz J, Ramírez-Tejero JA, Fernández-Pozo N, Leyva-Pérez M de la O, Yan H, Rosa R de la, et al. Transposon activation is a major driver in the genome evolution of cultivated olive trees ( *Olea europaea* L.). Plant Genome. 2020;:e20010. doi:10.1002/tpg2.20010. 10.1002/tpg2.20010 PMC12806974 33016633

[17] Besnard G, Hernández P, Khadari B, Dorado G, Savolainen V (2011). Genomic profiling of plastid DNA variation in the Mediterranean olive tree. BMC Plant Biol.

[18] Besnard G, El Bakkali A, Haouane H, Baali-Cherif D, Moukhli A, Khadari B (2013). Population genetics of Mediterranean and Saharan olives: geographic patterns of differentiation and evidence for early generations of admixture. Ann Bot.

[19] Haouane H, El Bakkali A, Moukhli A, Tollon C, Santoni S, Oukabli A (2011). Genetic structure and core collection of the World Olive Germplasm Bank of Marrakech: towards the optimised management and use of Mediterranean olive genetic resources. Genetica..

[20] Díez CM, Imperato A, Rallo L, Barranco D, Trujillo I (2012). Worldwide core collection of olive cultivars based on simple sequence repeat and morphological markers. Crop Sci.

[21] El Bakkali A, Haouane H, Moukhli A, Costes E, Van Damme P, Khadari B (2013). Construction of core collections suitable for association mapping to optimize use of Mediterranean olive (Olea europaea L.) genetic resources. PLoS One.

[22] Besnard G, Baradat P, Breton C, Khadari B, Bervillé A (2001). Olive domestication from structure of oleasters and cultivars using nuclear RAPDs and mitochondrial RFLPs. Genet Sel Evol.

[23] Cruz F, Julca I, Gómez-Garrido J, Loska D, Marcet-Houben M, Cano E (2016). Genome sequence of the olive tree, Olea europaea. Gigascience..

[24] Unver T, Wu Z, Sterck L, Turktas M, Lohaus R, Li Z (2017). Genome of wild olive and the evolution of oil biosynthesis. Proc Natl Acad Sci U S A.

[25] İpek A, İpek M, Ercişli S, Tangu NA (2017). Transcriptome-based SNP discovery by GBS and the construction of a genetic map for olive. Funct Integr Genomics.

[26] Julca I, Marcet-Houben M, Vargas P, Gabaldón T (2018). Phylogenomics of the olive tree (Olea europaea) reveals the relative contribution of ancient allo- and autopolyploidization events. BMC Biol.

[27] Camacho C, Coulouris G, Avagyan V, Ma N, Papadopoulos J, Bealer K (2009). BLAST+: architecture and applications. BMC Bioinformatics.

[28] de Abreu-Neto JB, Turchetto-Zolet AC, de Oliveira LFV, Zanettini MHB, Margis-Pinheiro M (2013). Heavy metal-associated isoprenylated plant protein (HIPP): characterization of a family of proteins exclusive to plants. FEBS J.

[29] Hundertmark M, Hincha DK (2008). LEA (late embryogenesis abundant) proteins and their encoding genes in *Arabidopsis thaliana*. BMC Genomics.

[30] Durner J, Shah J, Klessig DF (1997). Salicylic acid and disease resistance in plants. Trends Plant Sci.

[31] Pearce G, Moura DS, Stratmann J, Ryan CA (2001). RALF, a 5-kDa ubiquitous polypeptide in plants, arrests root growth and development. Proc Natl Acad Sci U S A.

[32] Vanholme R, Cesarino I, Rataj K, Xiao Y, Sundin L, Goeminne G (2013). Caffeoyl shikimate esterase (CSE) is an enzyme in the lignin biosynthetic pathway in Arabidopsis. Science..

[33] Liu Q, Luo L, Zheng L. Lignins: biosynthesis and biological functions in plants. Int J Mol Sci. 2018;19 10.3390/ijms19020335. 10.3390/ijms19020335 PMC5855557 29364145

[34] Ashikawa I, Abe F, Nakamura S (2013). DOG1-like genes in cereals: investigation of their function by means of ectopic expression in Arabidopsis. Plant Sci.

[35] Rashid A, Deyholos MK (2011). PELPK1 (At5g09530) contains a unique pentapeptide repeat and is a positive regulator of germination in Arabidopsis thaliana. Plant Cell Rep.

[36] He S, Tan G, Liu Q, Huang K, Ren J, Zhang X (2011). The LSD1-interacting protein GILP is a LITAF domain protein that negatively regulates hypersensitive cell death in Arabidopsis. PLoS One.

[37] Lin R, Wang H (2004). Arabidopsis FHY3/FAR1 gene family and distinct roles of its members in light control of Arabidopsis development. Plant Physiol.

[38] Wang H, Wang H (2015). Multifaceted roles of FHY3 and FAR1 in light signaling and beyond. Trends Plant Sci.

[39] Mariotti R, Cultrera NGM, Díez CM, Baldoni L, Rubini A (2010). Identification of new polymorphic regions and differentiation of cultivated olives (Olea europaea L.) through plastome sequence comparison. BMC Plant Biol.

[40] Van de Paer C, Bouchez O, Besnard G (2018). Prospects on the evolutionary mitogenomics of plants: a case study on the olive family (Oleaceae). Mol Ecol Resour.

[41] Huang J-L, Sun G-L, Zhang D-M (2010). Molecular evolution and phylogeny of the angiosperm ycf2 gene. J Syst Evol.

[42] Drescher A, Ruf S, Calsa T, Carrer H, Bock R (2000). The two largest chloroplast genome-encoded open reading frames of higher plants are essential genes. Plant J.

[43] Wicke S, Schneeweiss GM, dePamphilis CW, Müller KF, Quandt D (2011). The evolution of the plastid chromosome in land plants: gene content, gene order, gene function. Plant Mol Biol.

[44] Besnard G, Henry P, Wille L, Cooke D, Chapuis E (2007). On the origin of the invasive olives (Olea europaea L., Oleaceae). Heredity..

[45] Besnard G, Khadari B, Villemur P, Bervillé A (2000). Cytoplasmic male sterility in the olive (Olea europaea L.). Theor Appl Genet.

[46] Fehrer J, Gemeinholzer B, Chrtek J, Bräutigam S (2007). Incongruent plastid and nuclear DNA phylogenies reveal ancient intergeneric hybridization in Pilosella hawkweeds (Hieracium, Cichorieae, Asteraceae). Mol Phylogenet Evol.

[47] Barber JC, Finch CC, Francisco-Ortega J, Santos-Guerra A, Jansen RK (2007). Hybridization in Macaronesian Sideritis (Lamiaceae): evidence from incongruence of multiple independent nuclear and chloroplast sequence datasets. Taxon..

[48] Pelser PB, Kennedy AH, Tepe EJ, Shidler JB, Nordenstam B, Kadereit JW (2010). Patterns and causes of incongruence between plastid and nuclear Senecioneae (Asteraceae) phylogenies. Am J Bot.

[49] Green RE, Krause J, Briggs AW, Maricic T, Stenzel U, Kircher M (2010). A draft sequence of the Neandertal genome. Science..

[50] Malinsky M. Dsuite - fast D-statistics and related admixture evidence from VCF files. BioRxiv. 2019; 10.1101/634477. 10.1111/1755-0998.13265 PMC7116594 33012121

[51] Lumaret R, Ouazzani N, Michaud H, Vivier G, Deguilloux MF, Di Giusto F (2004). Allozyme variation of oleaster populations (wild olive tree) (Olea europaea L.) in the Mediterranean Basin. Heredity..

[52] Doebley JF, Gaut BS, Smith BD (2006). The molecular genetics of crop domestication. Cell..

[53] McKey D, Elias M, Pujol B, Duputié A (2010). The evolutionary ecology of clonally propagated domesticated plants. New Phytol.

[54] Gaut BS, Díez CM, Morrell PL (2015). Genomics and the contrasting dynamics of annual and perennial domestication. Trends Genet.

[55] Terhorst J, Kamm JA, Song YS (2017). Robust and scalable inference of population history from hundreds of unphased whole genomes. Nat Genet.

[56] Klepo T, Toumi A, De La Rosa R, LeÓn L, Belaj A (2014). Agronomic evaluation of seedlings from crosses between the main Spanish olive cultivar ‘Picual’ and two wild olive trees. J Hortic Sci Biotechnol.

[57] Chu Y, Su X, Huang Q, Zhang X (2009). Patterns of DNA sequence variation at candidate gene loci in black poplar (*Populus nigra* L.) as revealed by single nucleotide polymorphisms. Genetica..

[58] Tuskan GA, Difazio S, Jansson S, Bohlmann J, Grigoriev I, Hellsten U (2006). The genome of black cottonwood, *Populus trichocarpa* (Torr. & Gray). Science..

[59] Biswas S, Akey JM (2006). Genomic insights into positive selection. Trends Genet.

[60] Pavlidis P, Živkovic D, Stamatakis A, Alachiotis N (2013). SweeD: likelihood-based detection of selective sweeps in thousands of genomes. Mol Biol Evol.

[61] Kaya HB, Akdemir D, Lozano R, Cetin O, Sozer Kaya H, Sahin M (2019). Genome wide association study of 5 agronomic traits in olive (Olea europaea L.). Sci Rep.

[62] Luu K, Bazin E, Blum MGB (2017). pcadapt: an R package to perform genome scans for selection based on principal component analysis. Mol Ecol Resour.

[63] Foll M, Gaggiotti O (2008). A genome-scan method to identify selected loci appropriate for both dominant and codominant markers: a Bayesian perspective. Genetics..

[64] Burgarella C, Barnaud A, Kane NA, Jankowski F, Scarcelli N, Billot C (2019). Adaptive introgression: an untapped evolutionary mechanism for crop adaptation. Front Plant Sci.

[65] Zhou Y, Minio A, Massonnet M, Solares E, Lv Y, Beridze T (2019). The population genetics of structural variants in grapevine domestication. Nat Plants..

[66] Baldoni L, Tosti N, Ricciolini C, Belaj A, Arcioni S, Pannelli G (2006). Genetic structure of wild and cultivated olives in the Central Mediterranean basin. Ann Bot.

[67] Davis PH (1978). Flora of Turkey.

[68] Lumaret R, Ouazzani N (2001). Plant genetics. Ancient wild olives in Mediterranean forests Nature.

[69] Besnard G, Terral J-F, Cornille A (2018). On the origins and domestication of the olive: a review and perspectives. Ann Bot.

[70] Vargas P, Talavera S (2012). Olea. Flora Ibérica.

[71] Gaut BS, Seymour DK, Liu Q, Zhou Y (2018). Demography and its effects on genomic variation in crop domestication. Nat Plants.

[72] Zhou Y, Massonnet M, Sanjak JS, Cantu D, Gaut BS (2017). Evolutionary genomics of grape (*Vitis vinifera ssp. vinifera*) domestication. Proc Natl Acad Sci U S A.

[73] Bartolini G, Petruccelli R (2002). Classification, origin, diffusion and history of the olive.

[74] Massei G, Hartley SE (2000). Disarmed by domestication? Induced responses to browsing in wild and cultivated olive. Oecologia..

[75] Tang H, Zhang X, Miao C, Zhang J, Ming R, Schnable JC (2015). ALLMAPS: robust scaffold ordering based on multiple maps. Genome Biol.

[76] Li H, Durbin R (2009). Fast and accurate short read alignment with burrows-wheeler transform. Bioinformatics..

[77] Conesa A, Götz S, García-Gómez JM, Terol J, Talón M, Robles M (2005). Blast2GO: a universal tool for annotation, visualization and analysis in functional genomics research. Bioinformatics..

[78] Altschul SF, Madden TL, Schäffer AA, Zhang J, Zhang Z, Miller W (1997). Gapped BLAST and PSI-BLAST: a new generation of protein database search programs. Nucleic Acids Res.

[79] Jones P, Binns D, Chang H-Y, Fraser M, Li W, McAnulla C (2014). InterProScan 5: genome-scale protein function classification. Bioinformatics..

[80] Waskom M, Botvinnik O, O’Kane D, Hobson P, Lukauskas S, Gemperline DC, et al. Mwaskom/Seaborn: V0.8.1 (September 2017). Zenodo. 2017. doi:10.5281/zenodo.883859.

[81] Dierckxsens N, Mardulyn P, Smits G (2017). NOVOPlasty: de novo assembly of organelle genomes from whole genome data. Nucleic Acids Res.

[82] Bankevich A, Nurk S, Antipov D, Gurevich AA, Dvorkin M, Kulikov AS (2012). SPAdes: a new genome assembly algorithm and its applications to single-cell sequencing. J Comput Biol.

[83] Boetzer M, Henkel CV, Jansen HJ, Butler D, Pirovano W (2011). Scaffolding pre-assembled contigs using SSPACE. Bioinformatics..

[84] Boetzer M, Pirovano W (2012). Toward almost closed genomes with GapFiller. Genome Biol.

[85] Slater GSC, Birney E (2005). Automated generation of heuristics for biological sequence comparison. BMC Bioinformatics..

[86] Lowe TM, Chan PP (2016). tRNAscan-SE on-line: integrating search and context for analysis of transfer RNA genes. Nucleic Acids Res.

[87] McKenna A, Hanna M, Banks E, Sivachenko A, Cibulskis K, Kernytsky A (2010). The genome analysis toolkit: a MapReduce framework for analyzing next-generation DNA sequencing data. Genome Res.

[88] Danecek P, Auton A, Abecasis G, Albers CA, Banks E, DePristo MA (2011). The variant call format and VCFtools. Bioinformatics..

[89] Evanno G, Regnaut S, Goudet J (2005). Detecting the number of clusters of individuals using the software STRUCTURE: a simulation study. Mol Ecol.

[90] Earl DA, vonHoldt BM (2012). STRUCTURE HARVESTER: a website and program for visualizing STRUCTURE output and implementing the Evanno method. Conserv Genet Resour.

[91] Rosenberg NA (2004). DISTRUCT: a program for the graphical display of population structure. Mol Ecol Notes.

[92] Purcell S, Neale B, Todd-Brown K, Thomas L, Ferreira MAR, Bender D (2007). PLINK: a tool set for whole-genome association and population-based linkage analyses. Am J Hum Genet.

[93] Katoh K, Kuma K, Toh H, Miyata T (2005). MAFFT version 5: improvement in accuracy of multiple sequence alignment. Nucleic Acids Res.

[94] Capella-Gutiérrez S, Silla-Martínez JM, Gabaldón T (2009). trimAl: a tool for automated alignment trimming in large-scale phylogenetic analyses. Bioinformatics..

[95] Stamatakis A (2014). RAxML version 8: a tool for phylogenetic analysis and post-analysis of large phylogenies. Bioinformatics..

[96] Huson DH, Bryant D (2006). Application of phylogenetic networks in evolutionary studies. Mol Biol Evol.

[97] Benjamini Y, Hochberg Y (1995). Controlling the false discovery rate: a practical and powerful approach to multiple testing. J R Stat Soc Ser B Methodol.

[98] Quinlan AR, Hall IM (2010). BEDTools: a flexible suite of utilities for comparing genomic features. Bioinformatics..

[99] Xie Z, Wang L, Wang L, Wang Z, Lu Z, Tian D, et al. Mutation rate analysis via parent-progeny sequencing of the perennial peach. I. a low rate in woody perennials and a higher mutagenicity in hybrids. Proc Biol Sci. 2016;283 10.1098/rspb.2016.1016. 10.1098/rspb.2016.1016 PMC5095371 27798292

[100] Al-Shahrour F, Díaz-Uriarte R, Dopazo J (2004). FatiGO: a web tool for finding significant associations of gene ontology terms with groups of genes. Bioinformatics..

[101] Abascal F, Corvelo A, Cruz F, Villanueva-Cañas JL, Vlasova A, Marcet-Houben M (2016). Extreme genomic erosion after recurrent demographic bottlenecks in the highly endangered Iberian lynx. Genome Biol.

[102] McDonald JH, Kreitman M (1991). Adaptive protein evolution at the Adh locus in drosophila. Nature..

[103] Nielsen R, Williamson S, Kim Y, Hubisz MJ, Clark AG, Bustamante C (2005). Genomic scans for selective sweeps using SNP data. Genome Res.

