# Supplementary material for: Genomic evidence for recurrent genetic admixture during the domestication of Mediterranean olive trees (Olea europaea L.)
Source: BMC Biol. 2020 Oct 26;18:148. doi: 10.1186/s12915-020-00881-6 (PMC7586694; doi:10.1186/s12915-020-00881-6)
Supplement: Supplementary file 1 — Additional file 1 Fig. S1. Genome comparison of sylvestris and europaea. (a) Cumulative genome length per scaffold ranked in order of size for the genome assembly of europaea (Oe9 - green, Oe6 - olive, picual - orange) and sylvestris (blue). A straight vertical line represents a perfect genome assembly. The horizontal plateaus indicate many small scaffolds. The top right end of each curve shows the total number of scaffolds. (b) Syntenic plot of the genome of europaea against sylvestris generated by SynMap. (c) Histogram of log 10 transformed Ks values of syntenic gene pairs identified as calculated by SynMap. Fig. S2. Plastid genome of the cultivar Farga. Protein coding genes are shown in green, rRNAs in light blue, and tRNAs in purple. The SNPs are shown per each individual sequenced in this study in the following order starting from outside: ‘Arbequina’, ‘Picual’, ‘Beladi’, ‘Sorani’, ‘Koroneiki’, ‘Frantoio’, ‘Lechin de Granada’, ‘Lechin de Sevilla’, ‘Megaritiki’, ‘Chemlal de Kabilye’, sylvestris-T, sylvestris-S. Fig. S3. Mitochondrial genome of the cultivar Farga. Protein coding genes are shown in green, rRNAs in light blue, and tRNAs in purple. The SNPs are shown per each individual sequenced in this study in the following order starting from outside: ‘Arbequina’, ‘Picual’, ‘Beladi’, ‘Sorani’, ‘Koroneiki’, ‘Frantoio’, ‘Lechin de Granada’, ‘Lechin de Sevilla’, ‘Megaritiki’, ‘Chemlal de Kabilye’, sylvestris-T, sylvestris-S. Fig. S4. Homozygous SNP distribution along the nuclear genome. The SNPs are shown in windows of 100 Kb. Cultivars are plotted in green and sylvestris in blue. Since cv. Farga was used as a reference genome, we do not expect homozygous SNPs for this sample. Fig. S5. Heterozygous SNP distribution along the nuclear genome. The SNPs are shown in windows of 100 Kb. Cultivars are plotted in green and sylvestris in blue. Fig. S6. Principal component analysis (PCA) based on 24,724,756 nuclear SNPs. The first two principal components (PC1 and PC2) are p [file 12915_2020_881_MOESM1_ESM.pdf]

Figure S1

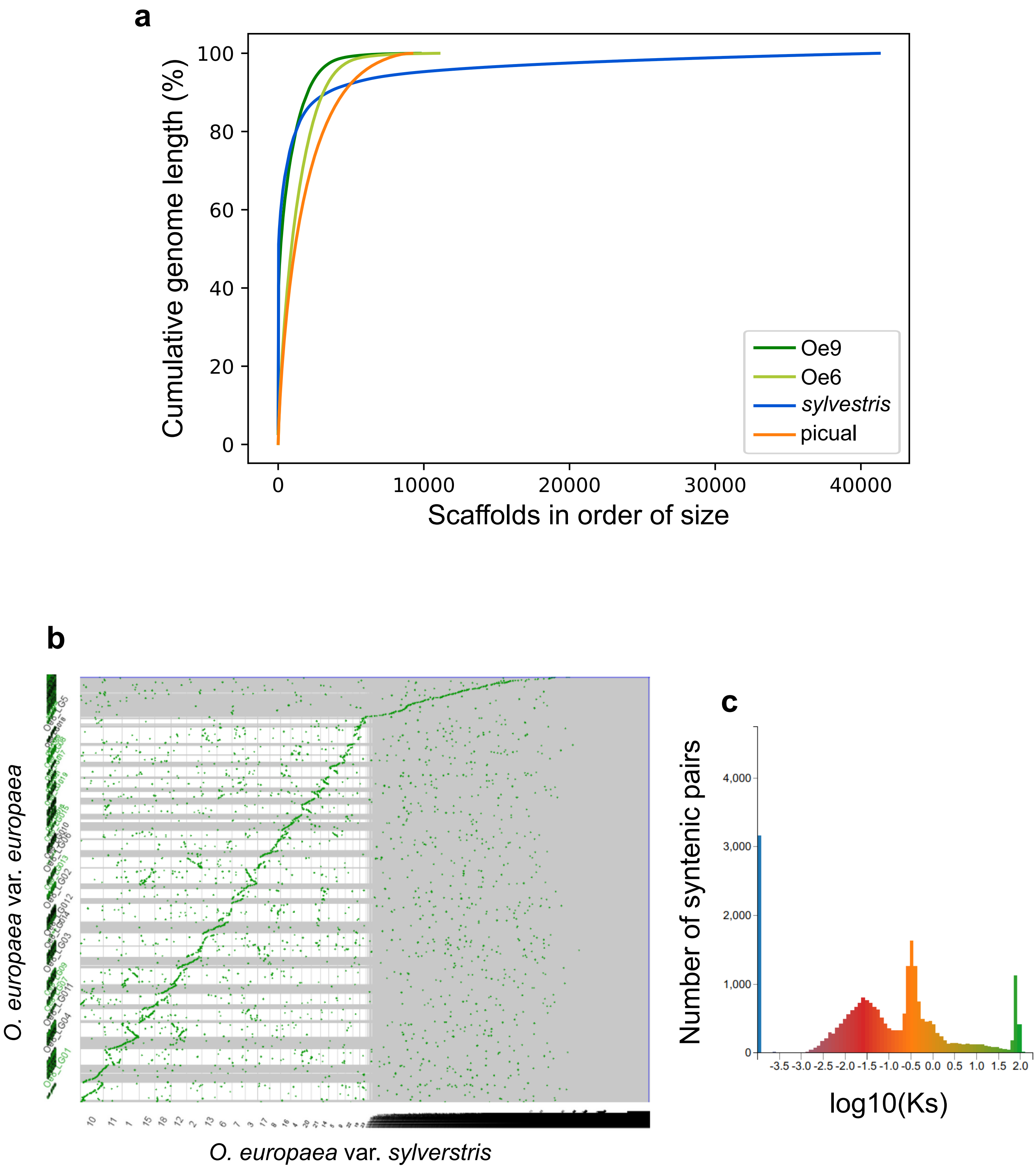
a
Cumulative genome length (%)
100
80
60
40
20
0
0
10000
20000
30000
40000
Scaffolds in order of size
Oe9
Oe6
sylvestris
picual
b
O. europaea var. europaea
O. europaea var. sylverstris
c
Number of syntenic pairs
4,000
3,000
2,000
1,000
0
-3.5 -3.0 -2.5 -2.0 -1.5 -1.0 -0.5 0.0 0.5 1.0 1.5 2.0
log10(Ks)

Figure S2

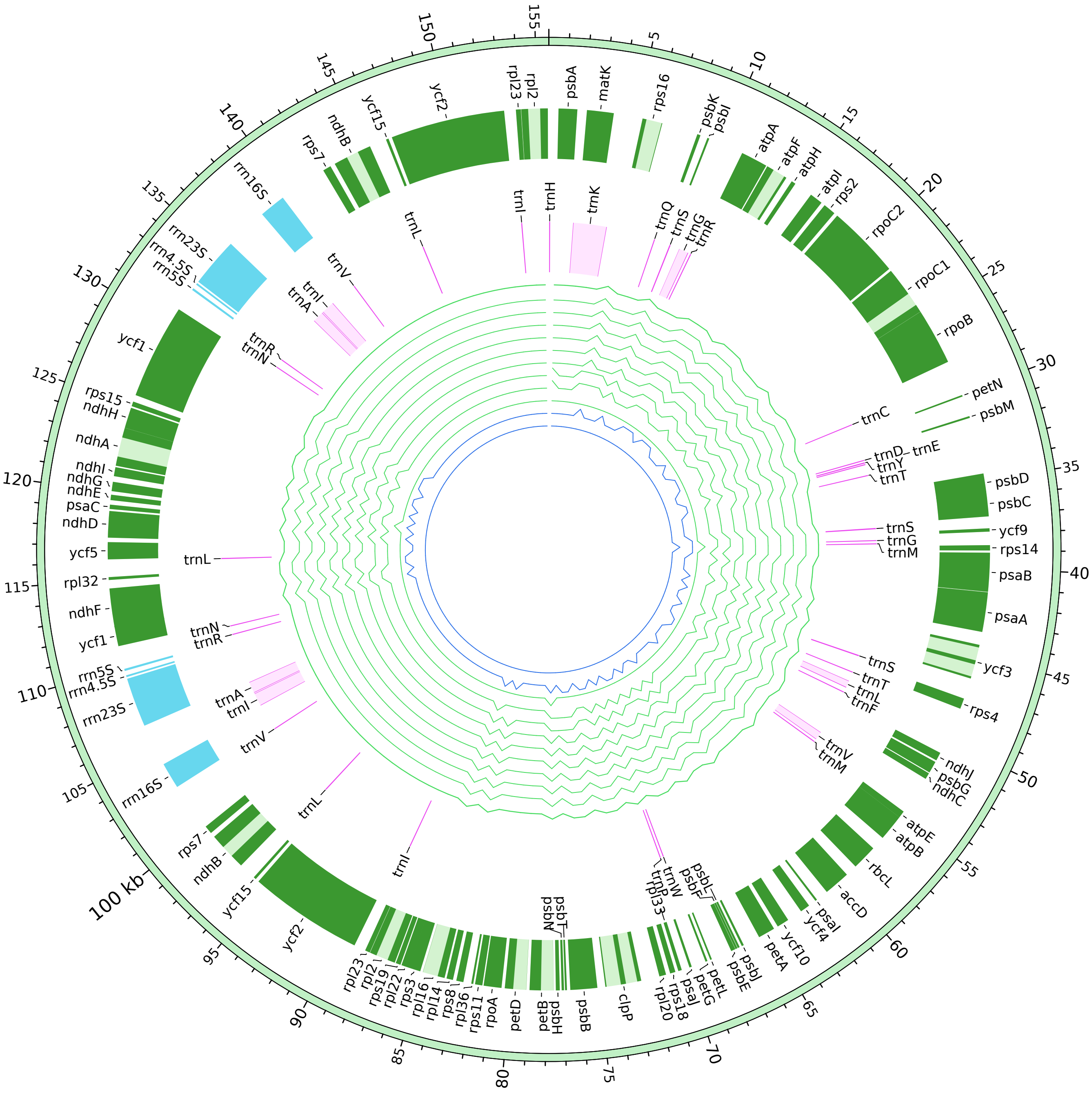

psbA
matK
rps16
psbK
psbI
atpA
atpF
atpH
atpI
rps2
rpoC2
rpoC1
rpoB
petN
psbM
psbD
psbC
ycf9
rps14
psaB
psaA
ycf3
rps4
ndhJ
psbG
ndhC
atpE
atpB
rbcL
accD
psaI
ycf4
ycf10
petA
psbJ
psbE
petL
petG
psaJ
rps18
rpl20
clpP
psbB
psbH
petB
petD
rpoA
rps11
rpl36
rps8
rpl14
rpl16
rps3
rpl22
rps19
rpl2
rpl23
ycf2
ycf15
ndhB
rps7
rrn16S
rrn23S
rrn4.5S
rrn5S
ycf1
ndhF
rpl32
ycf5
ndhD
psaC
ndhE
ndhG
ndhI
ndhA
ndhH
rps15
trnH
trnK
trnQ
trnS
trnG
trnR
trnC
trnD
trnY
trnE
trnT
trnM
trnL
trnF
trnV
trnW
trnP
rpl33
psbT
psbN
trnI
trnA
trnN
100 kb

Figure S3

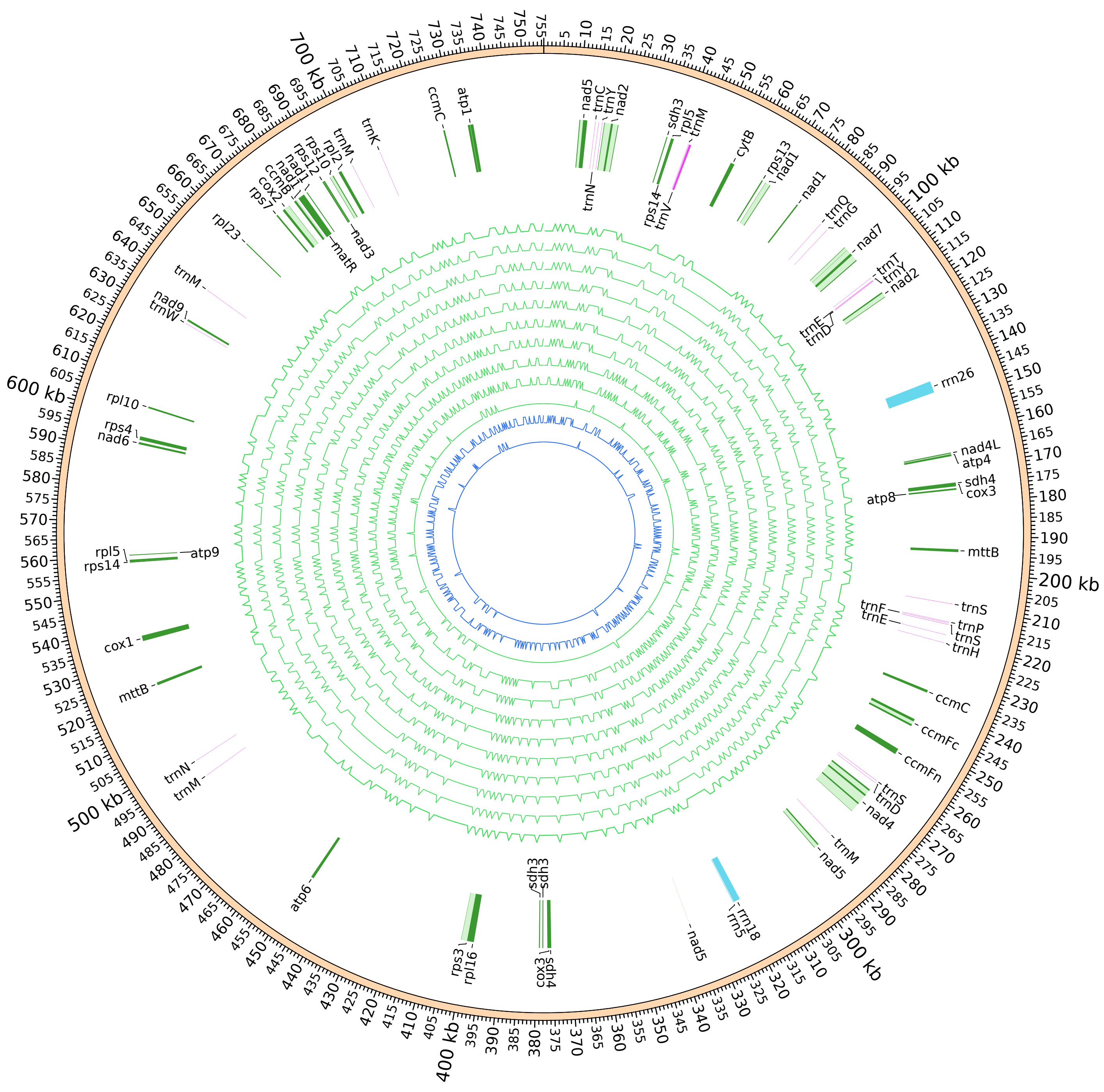

100 kb
200 kb
300 kb
400 kb
500 kb
600 kb
700 kb
nad5
trnC
trnY
nad2
trnN
sdh3
rpl5
trnM
rps14
trnV
cytB
rps13
nad1
nad1
trnQ
trnG
nad7
trnT
trnY
nad2
trnE
trnD
rrn26
nad4L
atp4
sdh4
cox3
atp8
mttB
trnS
trnF
trnP
trnE
trnS
trnH
ccmC
ccmFc
ccmFn
trnS
trnD
nad4
trnM
nad5
rrn18
rrn5
nad5
sdh3
sdh3
cox3
sdh4
rps3
rpl16
atp6
trnN
trnM
mttB
cox1
rpl5
rps14
atp9
rps4
nad6
rpl10
nad9
trnW
trnM
rpl23
rps7
cox2
ccmB
nad4
rps12
rps10
rpl2
trnM
nad3
matR
trnK
ccmC
atp1

Figure S4

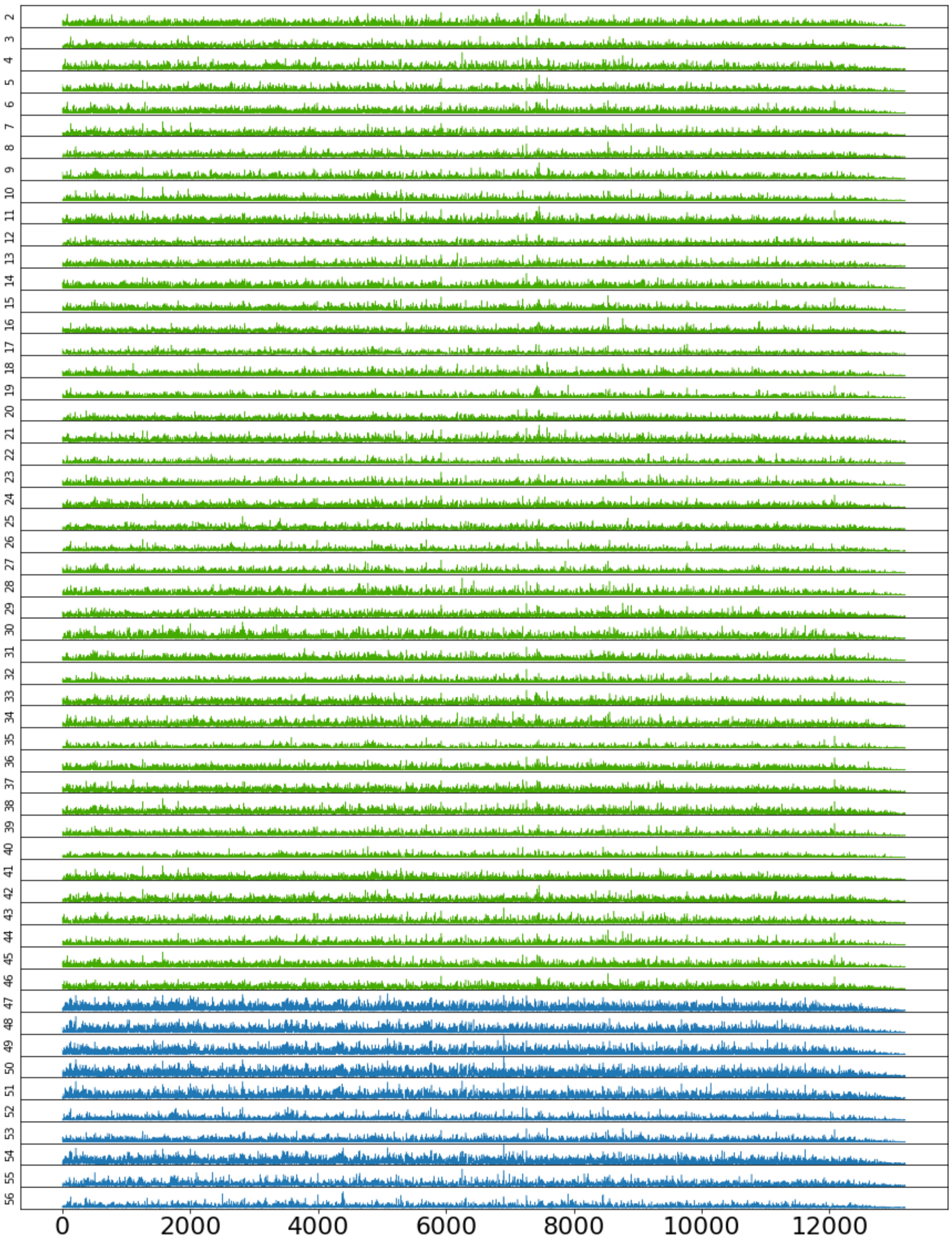

2 'Maarri'
3 'Koroneiki'
4 'Grappolo'
5 'Morrut'
6 'Abou Satl Mohazam'
7 'Barnea'
8 'Temprano'
9 'Myrtolia'
10 'Lechin de Sevilla'
11 'Sorani'
12 'Lianolia Kerkyras'
13 'Ocal'
14 'Fishomi'
15 'Manzanilla de Sevilla'
16 'Mavreya'
17 'Menya'
18 'Uslu'
19 'Lechin de Granada'
20 'Megaritiki'
21 'Beladi'
22 'Forastera de Tortosa'
23 'Royal'
24 'Hojiblanca'
25 'Piñonera'
26 'Frantoio'
27 'Arbequina'
28 'Leccino'
29 'Kalamon'
30 'Dokkar'
31 'Majhol-1013'
32 'Manzanillera de Huercal Overa'
33 'Jabali'
34 'Verdial de Velez-Malaga-1'
35 'Llumeta'
36 'Abou Kanani'
37 'Majhol-152'
38 'Mari'
39 'Picual'
40 'Chemlal de Kabilye'
41 'Zarza'
42 'Mastoidis'
43 'Klon-14-1812'
44 'Picudo'
45 'Abbadi Abou Gabra-842'
46 'Barri'
47 W7R224
48 W9R302
49 W11R37
50 sylvestris-P
51 W2R74
52 W8R225
53 sylvestris-T
54 W3R78
55 W1R198
56 W4R183

Figure S5

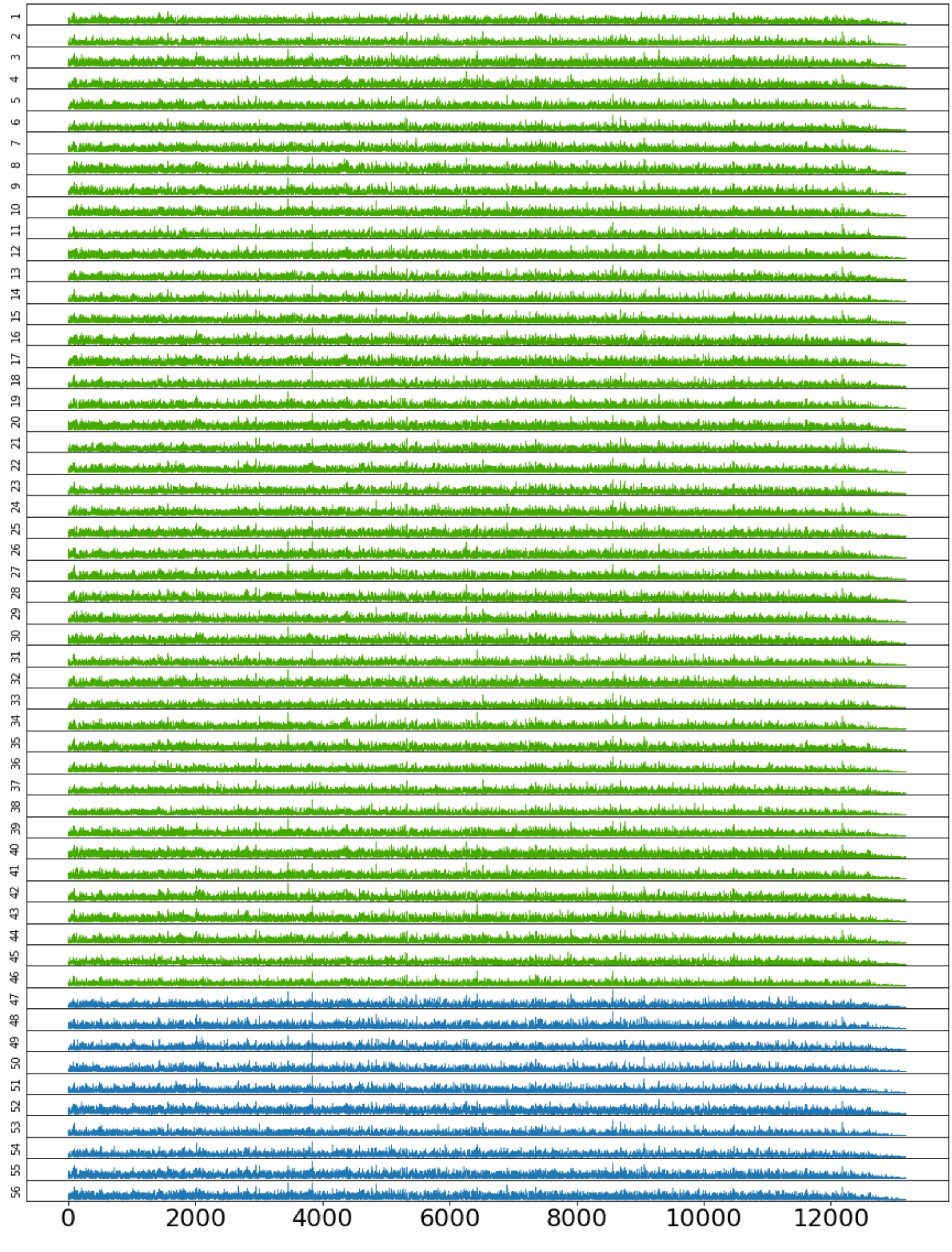

1 'Farga'
2 'Maarri'
3 'Koroneiki'
4 'Grappolo'
5 'Morrut'
6 'Abou Satl Mohazam'
7 'Barnea'
8 'Temprano'
9 'Myrtolia'
10 'Lechin de Sevilla'
11 'Sorani'
12 'Lianolia Kerkyras'
13 'Ocal'
14 'Fishomi'
15 'Manzanilla de Sevilla'
16 'Mavreya'
17 'Menya'
18 'Uslu'
19 'Lechin de Granada'
20 'Megaritiki'
21 'Beladi'
22 'Forastera de Tortosa'
23 'Royal'
24 'Hojiblanca'
25 'Piñonera'
26 'Frantoio'
27 'Arbequina'
28 'Leccino'
29 'Kalamon'
30 'Dokkar'
31 'Majhol-1013'
32 'Manzanillera de Huercal Overa'
33 'Jabali'
34 'Verdial de Velez-Malaga-1'
35 'Llumeta'
36 'Abou Kanani'
37 'Majhol-152'
38 'Mari'
39 'Picual'
40 'Chemlal de Kabilye'
41 'Zarza'
42 'Mastoidis'
43 'Klon-14-1812'
44 'Picudo'
45 'Abbadi Abou Gabra-842'
46 'Barri'
47 W7R224
48 W9R302
49 W11R37
50 sylvestris-P
51 W2R74
52 W8R225
53 sylvestris-T
54 W3R78
55 W1R198
56 W4R183

Figure S6

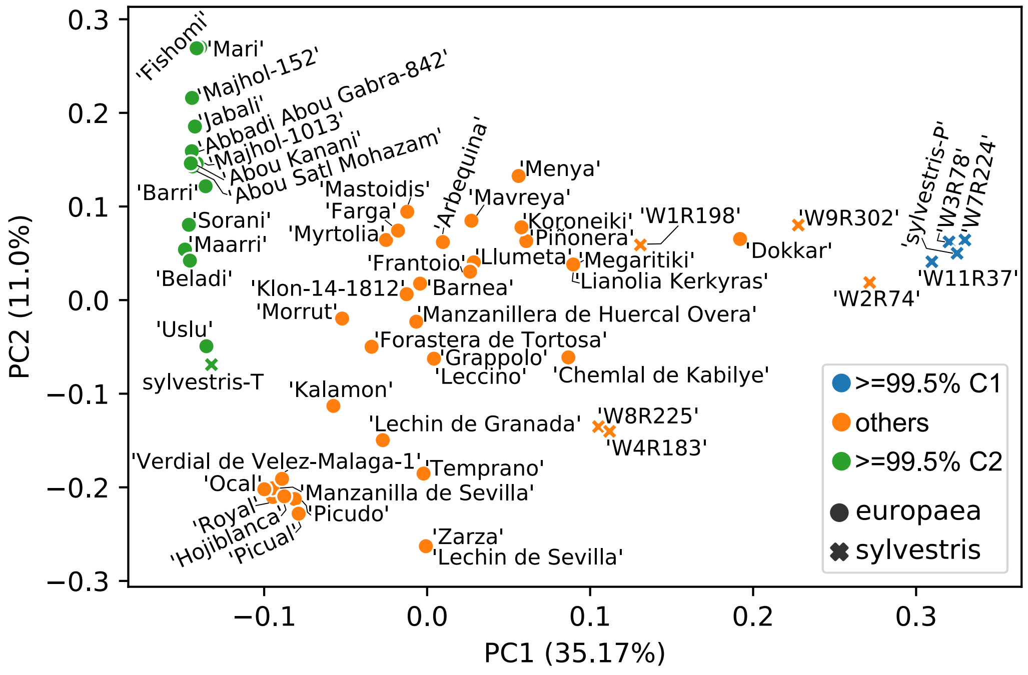

'Fishomi'
'Mari'
'Majhol-152'
'Jabali'
'Abbadi Abou Gabra-842'
'Majhol-1013'
'Abou Kanani'
'Abou Satl Mohazam'
'Barri'
'Sorani'
'Maarri'
'Beladi'
'Uslu'
sylvestris-T
'Mastoidis'
'Farga'
'Myrtolia'
'Arbequina'
'Menya'
'Mavreya'
'Koroneiki'
'Piñonera'
'W1R198'
'W9R302'
'Dokkar'
'Frantoio'
'Llumeta'
'Megaritiki'
'Lianolia Kerkyras'
'Klon-14-1812'
'Barnea'
'Morrut'
'Manzanillera de Huercal Overa'
'Forastera de Tortosa'
'Grappolo'
'Leccino'
'Chemlal de Kabilye'
'Kalamon'
'Lechin de Granada'
'W8R225'
'W4R183'
'Verdial de Velez-Malaga-1'
'Temprano'
'Ocal'
'Manzanilla de Sevilla'
'Royal'
'Hojiblanca'
'Picual'
'Picudo'
'Zarza'
'Lechin de Sevilla'
'sylvestris-P'
'W3R78'
'W7R224'
'W11R37'
'W2R74'
>=99.5% C1
others
>=99.5% C2
europaea
sylvestris
PC2 (11.0%)
PC1 (35.17%)

Figure S7

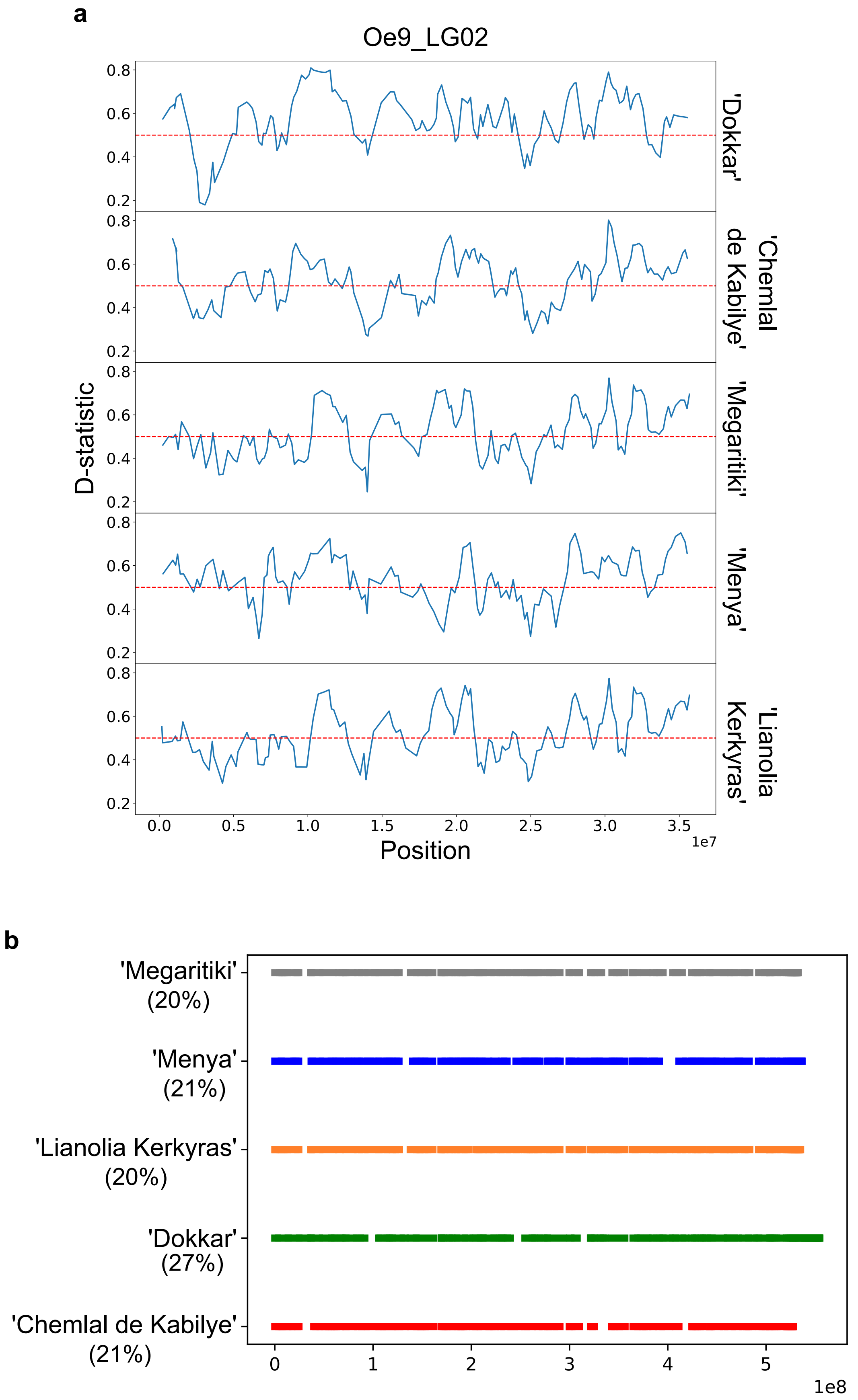

a
Oe9_LG02
D-statistic
0.2
0.4
0.6
0.8
'Dokkar'
'Chemlal de Kabilye'
'Megaritiki'
'Menya'
'Lianolia Kerkyras'
0.0
0.5
1.0
1.5
2.0
2.5
3.0
3.5
1e7
Position
b
'Megaritiki' (20%)
'Menya' (21%)
'Lianolia Kerkyras' (20%)
'Dokkar' (27%)
'Chemlal de Kabilye' (21%)
0
1
2
3
4
5
1e8

Figure S8

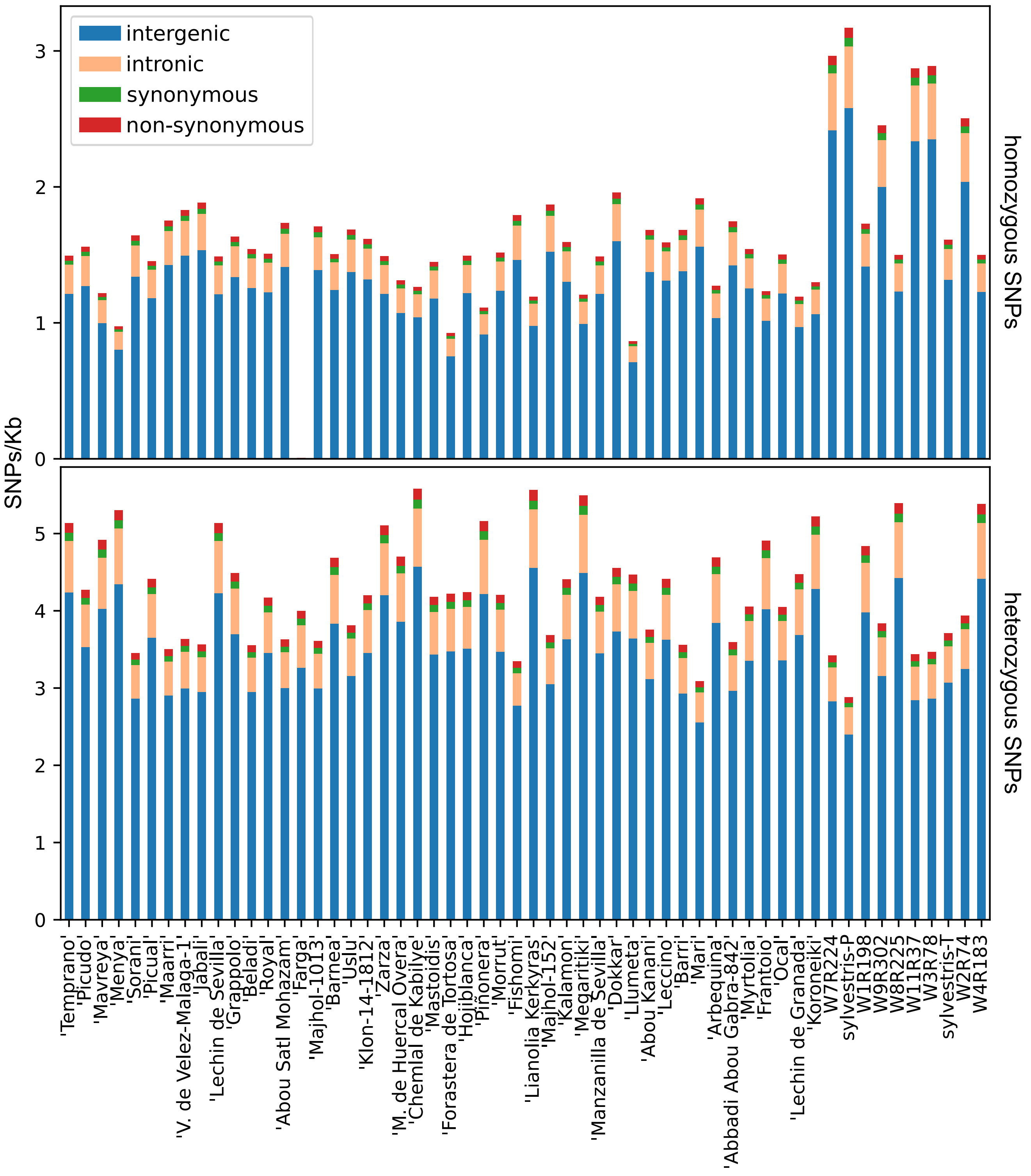

intergenic
intronic
synonymous
non-synonymous
SNPs/Kb
homozygous SNPs
heterozygous SNPs
0
1
2
3
4
5
'Temprano'
'Picudo'
'Mavreya'
'Menya'
'Sorani'
'Picual'
'Maarri'
'V. de Velez-Malaga-1'
'Jabali'
'Lechin de Sevilla'
'Grappolo'
'Beladi'
'Royal'
'Abou Satl Mohazam'
'Farga'
'Majhol-1013'
'Barnea'
'Uslu'
'Klon-14-1812'
'Zarza'
'M. de Huercal Overa'
'Chemlal de Kabilye'
'Mastoidis'
'Forastera de Tortosa'
'Hojiblanca'
'Piñonera'
'Morrut'
'Fishomi'
'Lianolia Kerkyras'
'Majhol-152'
'Kalamon'
'Megaritiki'
'Manzanilla de Sevilla'
'Dokkar'
'Llumeta'
'Abou Kanani'
'Leccino'
'Barri'
'Mari'
'Arbequina'
'Abbadi Abou Gabra-842'
'Myrtolia'
'Frantoio'
'Ocal'
'Lechin de Granada'
'Koroneiki'
W7R224
sylvestris-P
W1R198
W9R302
W8R225
W11R37
W3R78
sylvestris-T
W2R74
W4R183

Figure S9

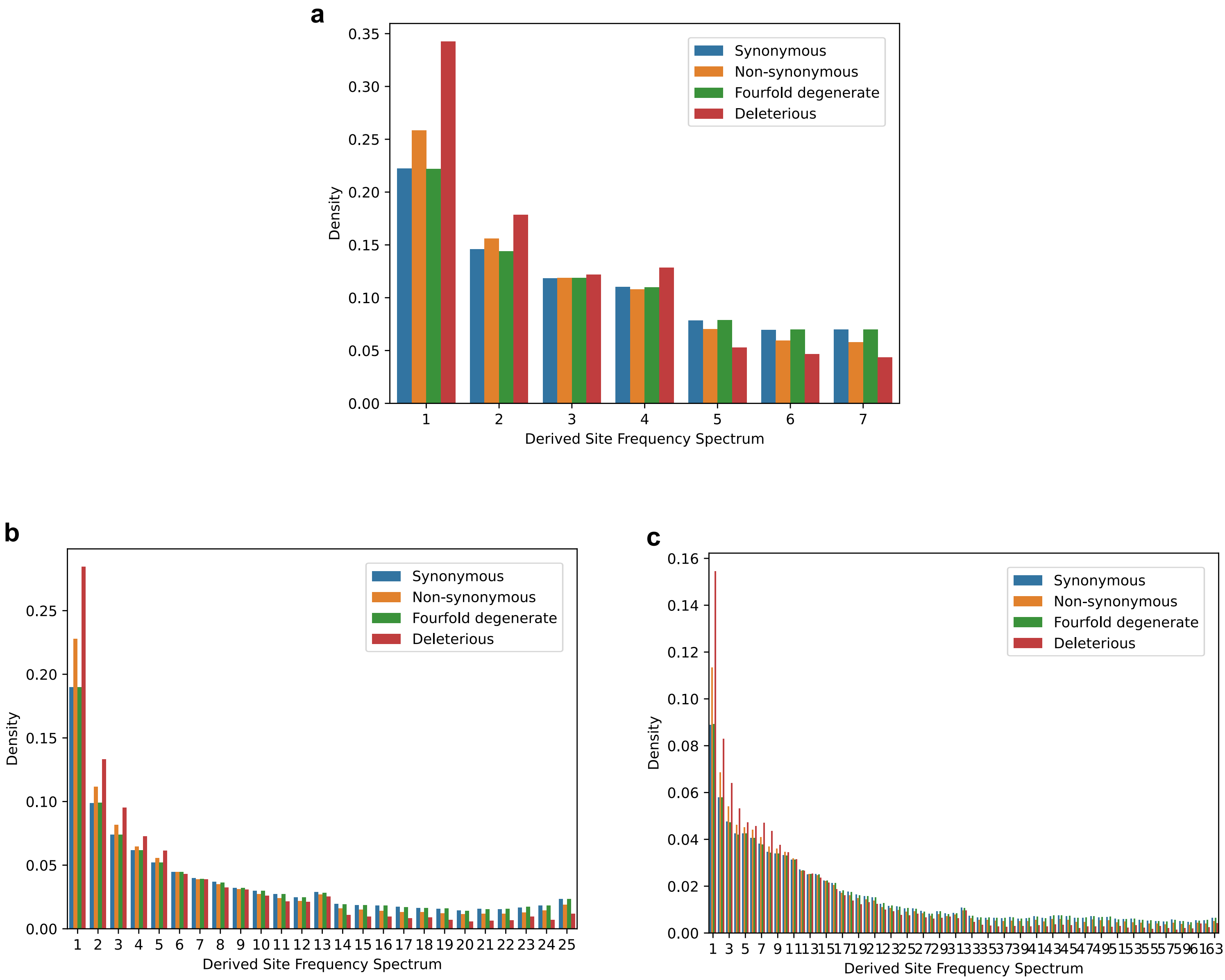
a
Synonymous
Non-synonymous
Fourfold degenerate
Deleterious
Density
0.00
0.05
0.10
0.15
0.20
0.25
0.30
0.35
1 2 3 4 5 6 7
Derived Site Frequency Spectrum
b
Synonymous
Non-synonymous
Fourfold degenerate
Deleterious
Density
0.00
0.05
0.10
0.15
0.20
0.25
1 2 3 4 5 6 7 8 9 10 11 12 13 14 15 16 17 18 19 20 21 22 23 24 25
Derived Site Frequency Spectrum
c
Synonymous
Non-synonymous
Fourfold degenerate
Deleterious
Density
0.00
0.02
0.04
0.06
0.08
0.10
0.12
0.14
0.16
Derived Site Frequency Spectrum
